# Supplementary material for: Identification of new interactors of eIF3f by endogenous proximity-dependent biotin labelling in human muscle cells
Source: Sci Rep. 2025 Dec 21;16:2812. doi: 10.1038/s41598-025-32702-7 (PMC12824170; doi:10.1038/s41598-025-32702-7)
Supplement: Supplementary file 1 — Supplementary Information. [file 41598_2025_32702_MOESM1_ESM.pdf]

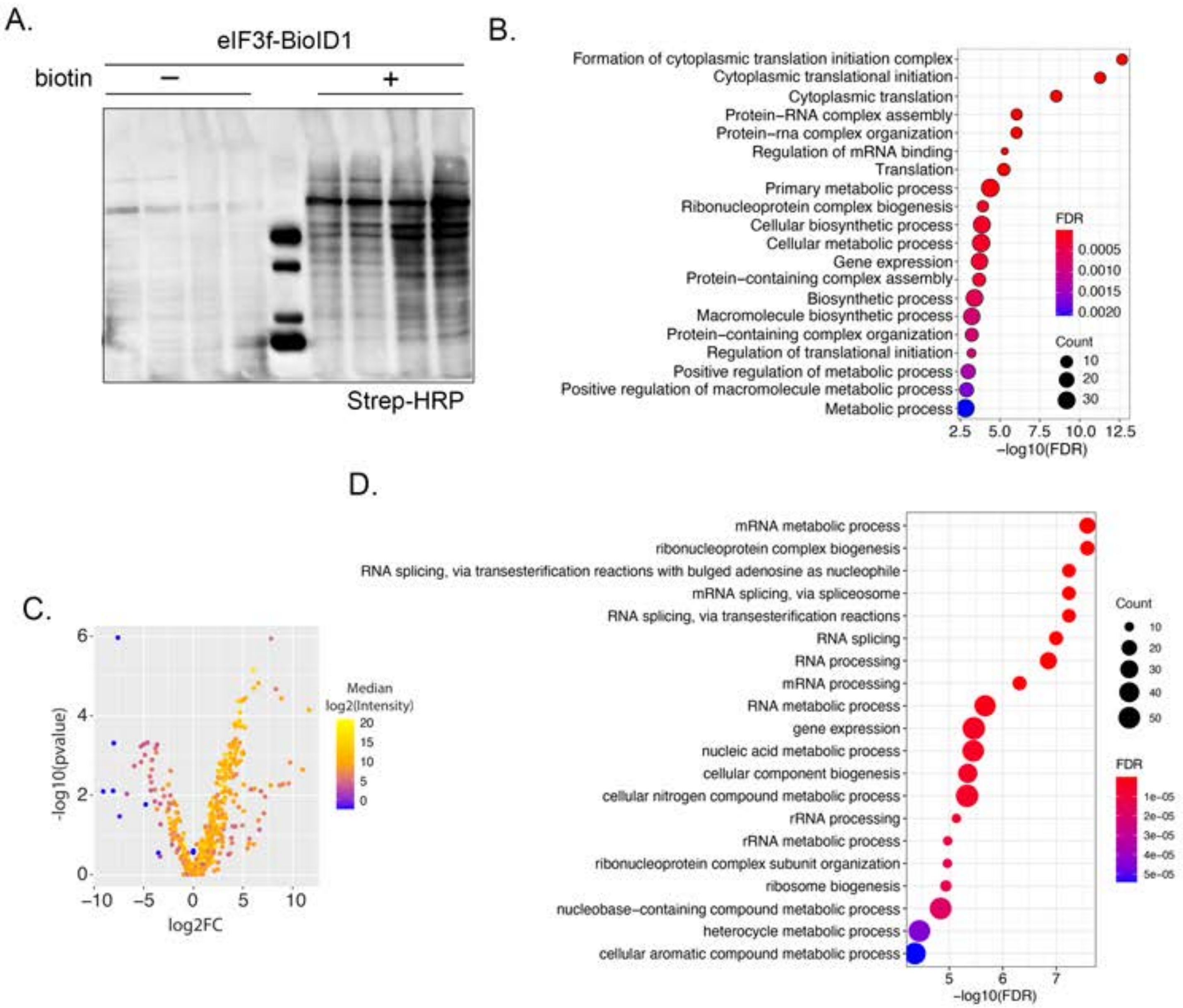

Supplementary Figure 1: Transient BioID1 biotinylation in HEK293 cells.
A. Streptavidin coupled HRP (abcam ≠ab7403) detection of biotin incorporation in lysates from HEK293 of overexpressing eIF3f-BioID1 and cultured 24hrs in presence or not (+/-) of 50µM biotin. The same lysates were further used for MS/LC coupled Streptavidin-dynabeads pull down experiment. Original blots are presented in Supplementary Figure 15. B. Functional annotation of proteins showing a significant enrichment after pulldown from HEK293 cell lysate expressing or not eIF3f_BioID1 in p resence of biotin. Only the top 20 pathways (based on FDR) are shown. C. Volcano plot distribution of the protein identified by MS/LC uponstreptavidin-dynabeads purification of biotinylated protein upon overexpression of BioID1 only proteinin HEK293 cells. D. Functional annotation of proteins showing a significant enrichment with BioID1 only protein. Only the top 20 pathways (based on FDR) are shown.

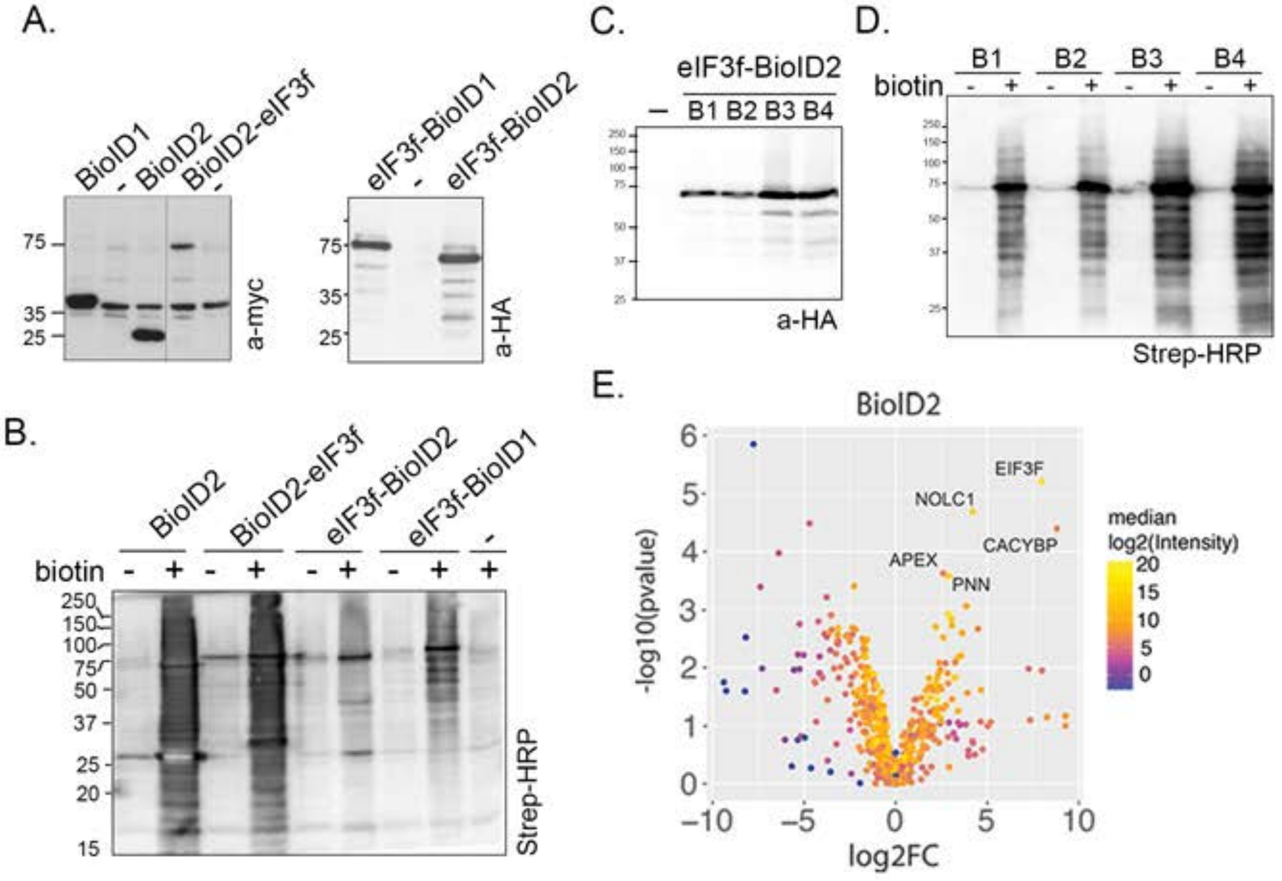

Supplementary Figure 2: BioID1 versus BioID2 C-terminal fusion chimera of eIF3f.

A.Transient expression in HEK293 cells of BioID1 and BioID2 fusion to the eIF3f protein (at C-ter or N-ter of eIF3f). Lysates obtained from triplicate transfections were separated on SDS gel and exogenously expressed protein detected with indicated antibody. Original blots are presented in Supplementary Figure 16. B. Cellular distribution of the different eIF3f-BioID1 ligase constructs expressed in A. C2C12 cells were transfected with the indicated constructs. N-terminally or C-terminally fused yellow fluorescent protein (YFP) to eIF3f protein was used as control. Original blots are presented in Supplementary Figure 17. C. Western blot detection of exogenously expressed eIF3f-BioID2 (B1 to B4) from HEK293 cell lysate used for MS/MS coupled Streptavidin-dynabeads pull down. Original blots are presented in Supplementary Figure 18. D. Streptavidin coupled HRP detection of biotin incorporation in lysates from HEK293 overexpressing BioID2 and cultured 24hrs in presence or not (+/-) of 50µM biotin. The same lysates were further used in E. We used the same protocol as previously described in figure 1. Original blots are presented in Supplementary Figure 19. E. Volcano plot distribution of the proteins identified by LC/MS upon Streptavidin-dynabeads purification of biotinylated proteins from transfected HEK cells (supplementary figure 1A) as described in A.

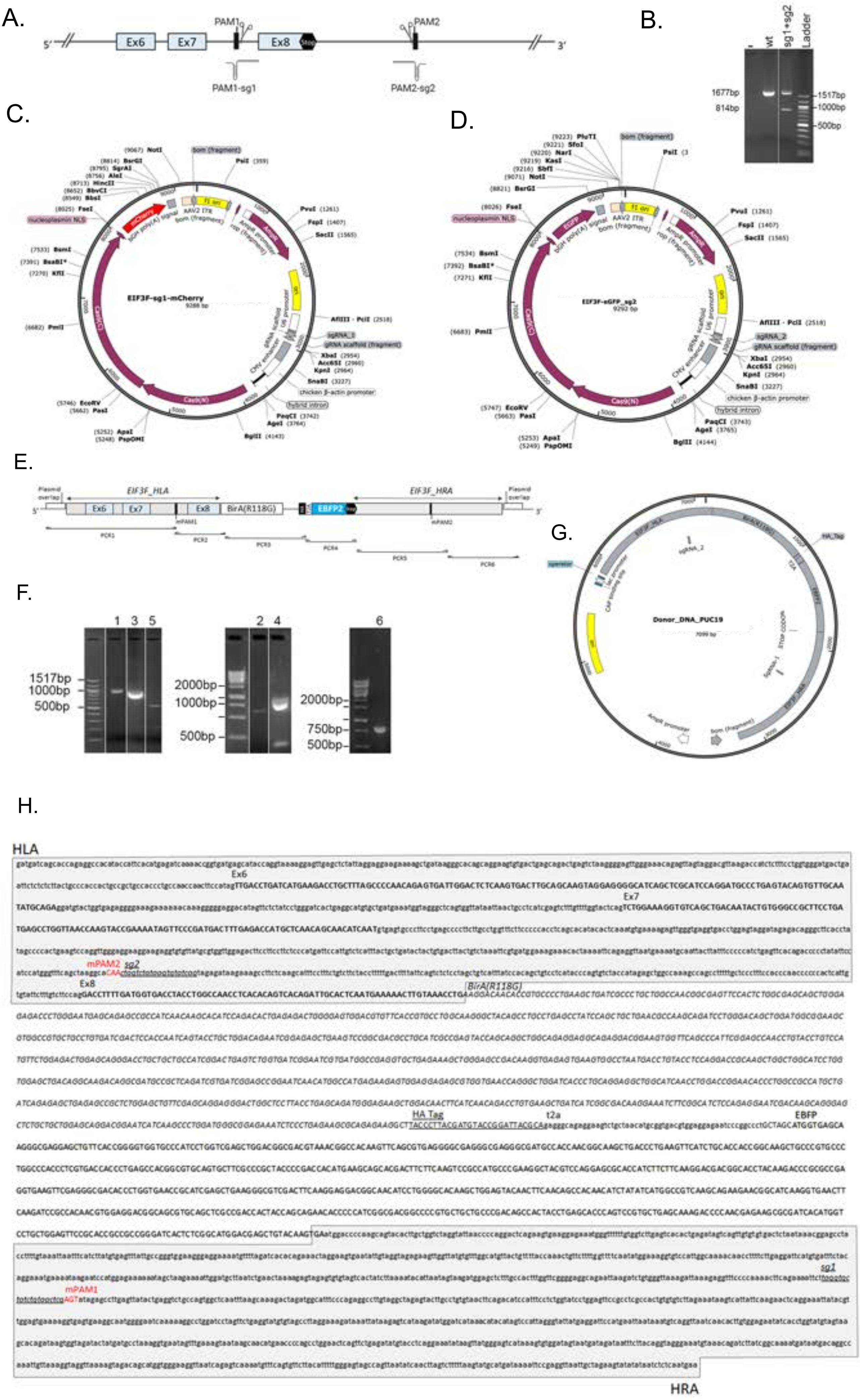

A.
5'
Ex6
Ex7
PAM1
Ex8
Stop
PAM2
3'
PAM1-sg1
PAM2-sg2
B.
-
wt
sg1+sg2
Ladder
1677bp
814bp
1517bp
1000bp
500bp
C.
EIF3F-sg1-mCherry
9288 bp
mCherry
nucleoplasmin NLS
bGH poly(A) signal
AAV2 ITR
f1 ori
bom (fragment)
rop (fragment)
AmpR promoter
AmpR
ori
U6 promoter
gRNA scaffold
sgRNA_1
gRNA scaffold (fragment)
CMV enhancer
chicken β-actin promoter
hybrid intron
Cas9(N)
Cas9(C)
D.
EIF3F-eGFP_sg2
9292 bp
EGFP
sgRNA_2
E.
EIF3F_HLA
EIF3F_HRA
Plasmid overlap
Ex6
Ex7
Ex8
BirA(R118G)
EBFP2
mPAM1
mPAM2
PCR1
PCR2
PCR3
PCR4
PCR5
PCR6
F.
1
3
5
2
4
6
1517bp
1000bp
500bp
2000bp
1000bp
500bp
2000bp
750bp
500bp
G.
Donor_DNA_PUC19
EIF3F_HLA
BirA(R118G)
HA_Tag
T2A
EBFP2
STOP CODON
sgRNA_1
sgRNA_2
EIF3F_HRA
AmpR promoter
bom (fragment)
ori
operator
H.
HLA
Ex6
Ex7
mPAM2
sg2
Ex8
BirA(R118G)
HA Tag
t2a
EBFP
sg1
mPAM1
HRA

Supplementary Figure 3: Cloning strategy and sequencing of EIF3F-BioID1 chimera.

A. Schematic representation of EIF3F gene 3'end displaying the recombination sites for PAM1-sg1 and PAM2-sg2 on both 5' and 3' ends of Exon8. B. Resolution on agarose gel of the fragment cleavage from sg1 to sg2 sites (814 BP). C. Annotation map of the EIF3f-sg1-mCherry plasmid (created with SnapGene). D. Annotation map of the EIF3f-eGFP_sg2 plasmid (created with SnapGene). E. Schematic representation of the donor DNA displaing right (RHA) and left homology arms (LHA) flanking the exon8-BioID1 and HA tagged EBFP inserts F. The Gibson cloning strategy was used to assemble the 6 different PCR amplicons of donor DNA in pUC.19 plasmid backbone. The amplified fragments corresponding to PCR1 to 6 were resolved on agarose gel before purification and assembly. G. Annotation map of the Donor DNA plasmid (created with SnapGene). H. Sequencing of the integrated construct. mPAM: mutated protospacer-adjacent motif; sg: single guide RNA; EBFP2: enhanced blue fluorescent protein; T2A: self-cleaving 2A peptide.

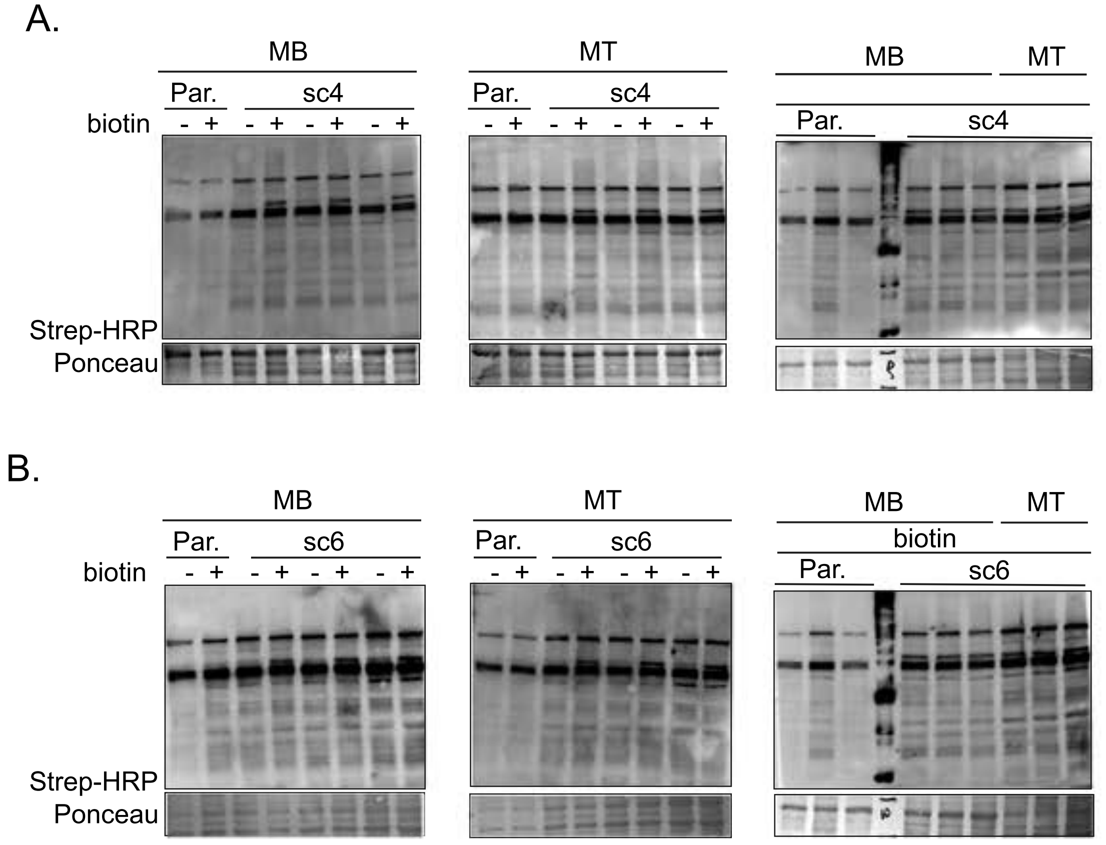

Supplementary Figure 4: Biotin incorporation in samples used for Streptavidin-dynabeads purification of eIF3f interactors.

A. Streptavidin coupled HRP detection of biotin incorporation form parental (Par.) and eIF3f-BioID1 knock-in sc4 lysates of MB or MT treated for 24hrs with 50µM biotin. The same lysates were further used for LC/MS coupled Streptavidin-dynabeads pull down experiment. Original blots are presented in Supplementary Figure 20. B. The same procedure as describe in A was conducted for sc6 lysate. Original blots are presented in Supplementary Figure 21.

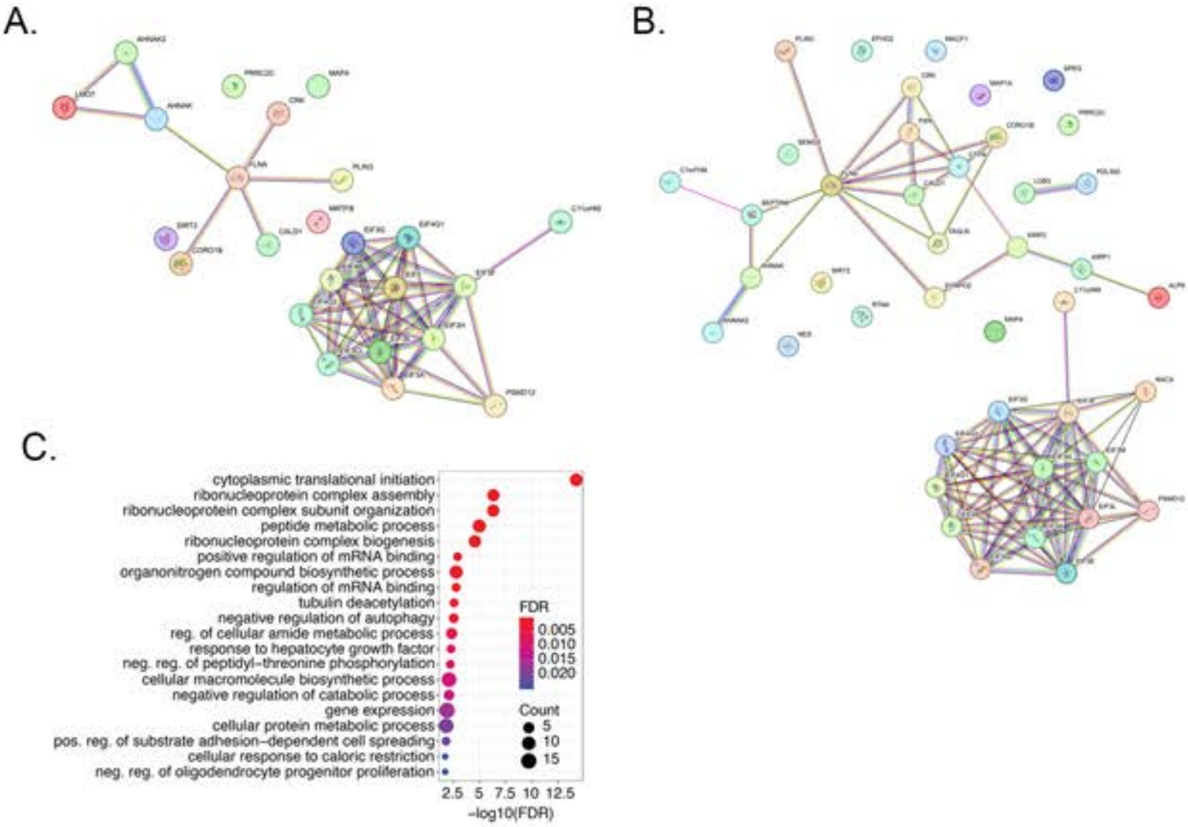

Supplementary Figure 5: Interaction map and functional enrichment of the eIF3f interactors identified in MB and MT.

A. Interaction map of LC/MS significantly identified ($p<0.01$ and $log2FC>0$) proteins from streptavidin-dynabeads pull-down experiment from MB (sc4/6) cultured in presence of biotin versus parental cells. B. Same as A obtained from MT (sc4/6) cells. C. Functional enrichment analysis ($p<0.01$ and $log2FC>0$) of the 21 conserved proteins identified as eIF3f binding partners in MB and MT (shown in figure 3F and 3G).

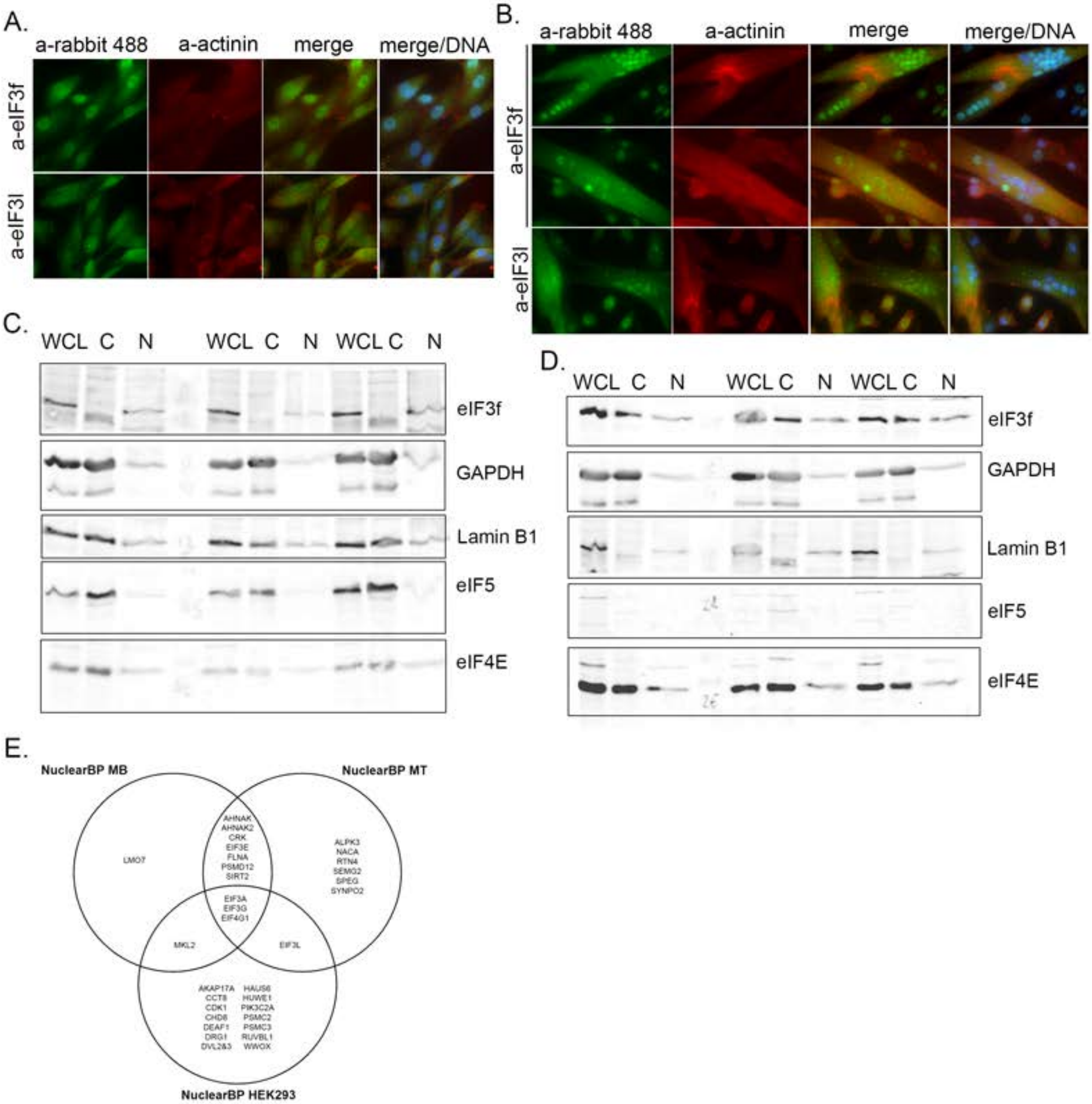

Supplementary Figure 6: Subcellular distribution of eIF3f protein and selected identified binding partners in MB and MT.

A. Immunodetection of eIF3 subunit F, and L (green) in parental myoblasts. Anti sarcomeric alpha-actinin-3 was visualised with Cy5 coupled anti mouse antibody and DNA was stained with Hoechst. B. The same immunodetection was conducted in myotubes. C. Whole cell lysate (WCL) or fractionated (C: cytoplasm and N: nuclear) lysates from parental MB were resolved on SDS/PAGE and probed with indicated antibodies. GAPDH and Lamin B1 were used respectively as cytoplasmic or nuclear markers. Signals from infrared (700nm) coupled secondary antibody were acquired with Odyssey (Li-Cor). Original blots are presented in Supplementary Figure 22. D. The same experiment as in C was conducted in sc4 MT. Original blots are presented in Supplementary Figure 23. E. Venn diagram of the nuclear binding partners (BP), according to https://www.genecards.org/nomenclature, identified for eIF3f-BioID1 chimaera in myoblasts, myotubes and HEK293 biotinylation experiments.

A.

Protein name	log2ratio_DSB	pValue_DSB
sp|P11279|LAMP1_HUMAN	5.46	9.16E-04
sp|O75112|LDB3_HUMAN	5.31	1.58E-03
sp|A4UGR9|XIRP2_HUMAN	5.2	4.84E-04
sp|P11055|MYH3_HUMAN	4.8	1.88E-03
sp|Q15772|SPEG_HUMAN	4.32	8.76E-04
sp|O00763|ACACB_HUMAN	3.92	8.34E-04
sp|Q702N8|XIRP1_HUMAN	3.86	6.95E-05
sp|P07686|HEXB_HUMAN	3.71	6.89E-04
sp|E9PAV3|NACAM_HUMAN	3.55	3.68E-04
sp|Q15050|RRS1_HUMAN	3.54	1.23E-03
sp|Q8WZ42|TITIN_HUMAN	3.54	7.88E-04
sp|Q9NQC3|RTN4_HUMAN	3.3	1.03E-03
sp|Q9NP64|NO40_HUMAN	3.2	3.39E-03
sp|Q9Y262|EIF3L_HUMAN	3.17	5.46E-04
sp|P13535|MYH8_HUMAN	2.98	2.88E-03
sp|P11182|ODB2_HUMAN	2.53	2.57E-03
sp|Q15361|TTF1_HUMAN	2.48	3.51E-03
sp|Q1ED39|KNOP1_HUMAN	2.17	2.87E-03
sp|P09651|ROA1_HUMAN	-1.73	3.55E-03
sp|Q9BR76|COR1B_HUMAN	-2.21	2.43E-03
sp|P16402|H13_HUMAN	-2.57	1.68E-03
sp|Q9H6J7|CK049_HUMAN	-2.69	8.59E-04
sp|Q5QNW6|H2B2F_HUMAN	-3.07	3.18E-03
sp|P0CJ85|DU4L2_HUMAN	-5.08	1.04E-03
sp|P62910|RL32_HUMAN	-7.77	1.42E-03

B.

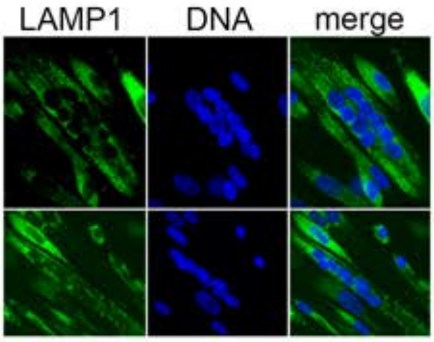

Supplementary Figure 7: "Core eIF3f-eIF3" interactors identified in MT and MB.
A. Table: list of interactors of the "core eIF3f-eIF3" identified in MT vs MB. Only proteins identified with high level of confidence are shown (min. med. int.>20; pVal<0.01). B. Immuno-detection of LAMP1 visualised with anti-rabbit Cy3 secondary antibody in parental differentiated (day 6) myotubes. DNA was stained with Hoechst.

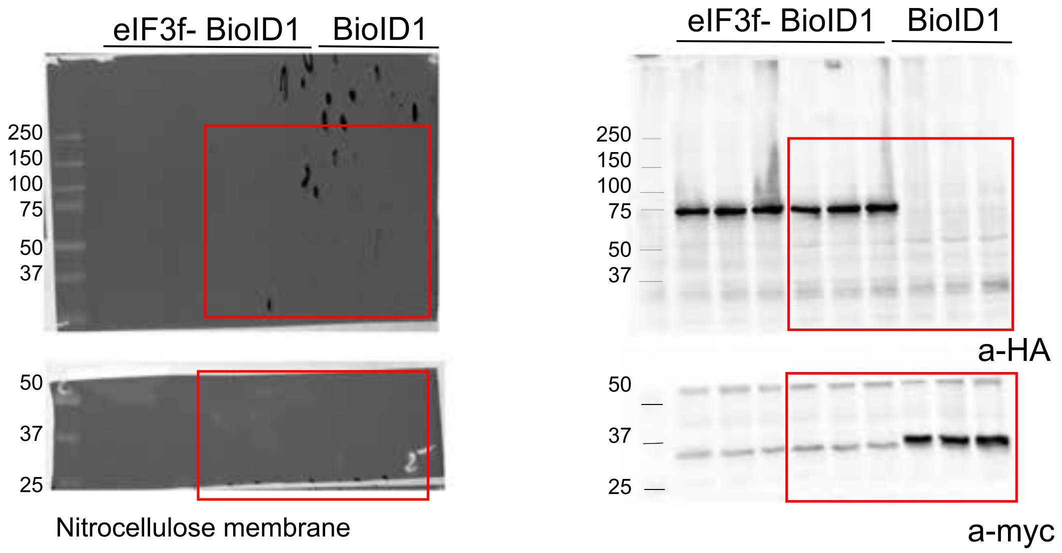

Supplementary Figure 8
Uncropped western blot for Figure 1B.

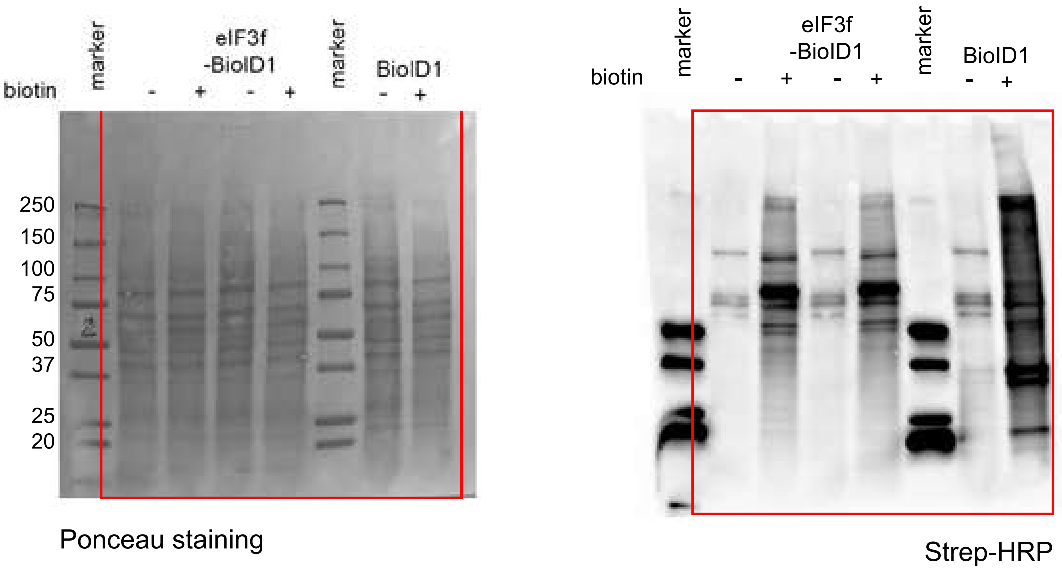

Supplementary Figure 9
Uncropped western blot for Figure 1C

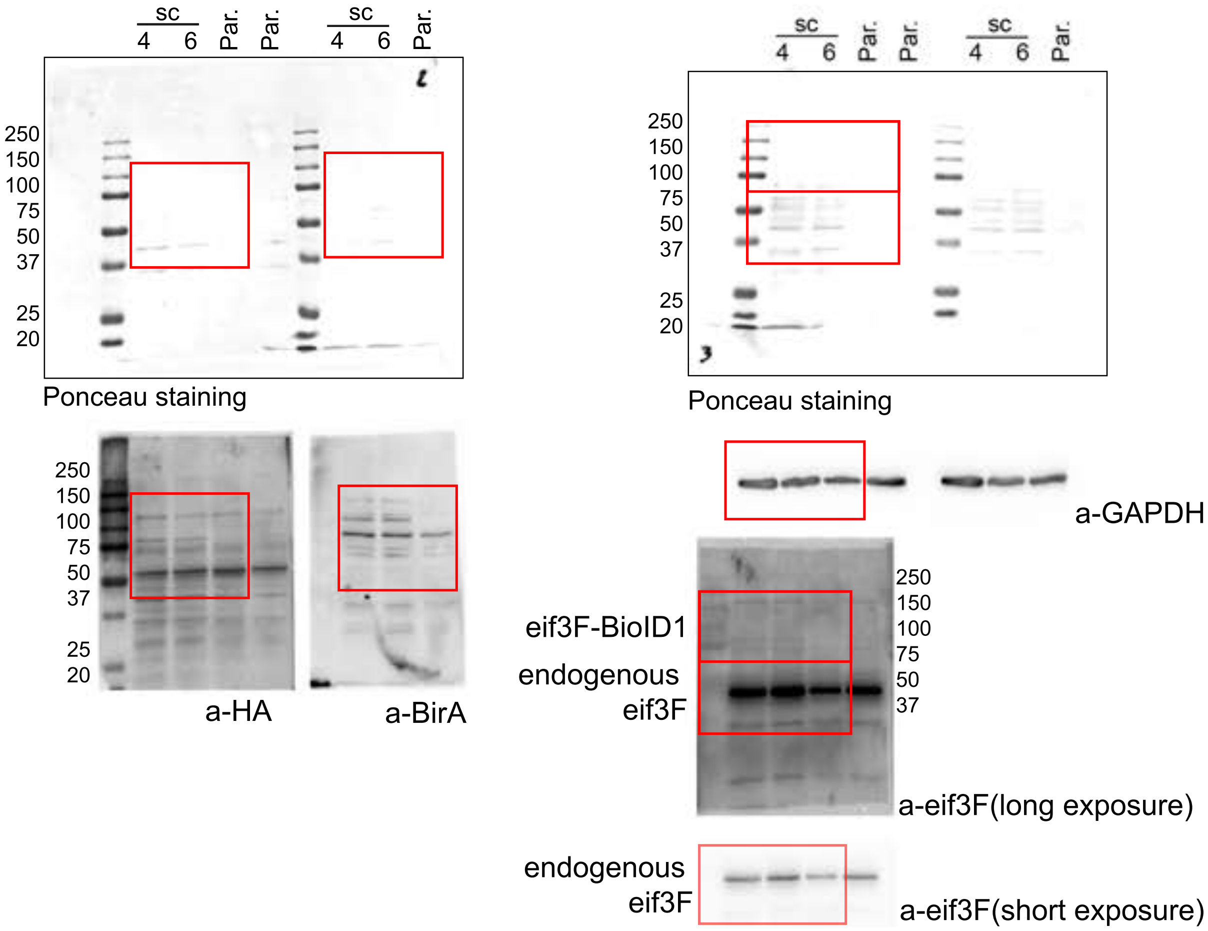

Supplementary Figure 10

Uncropped western blot for Figure 2D

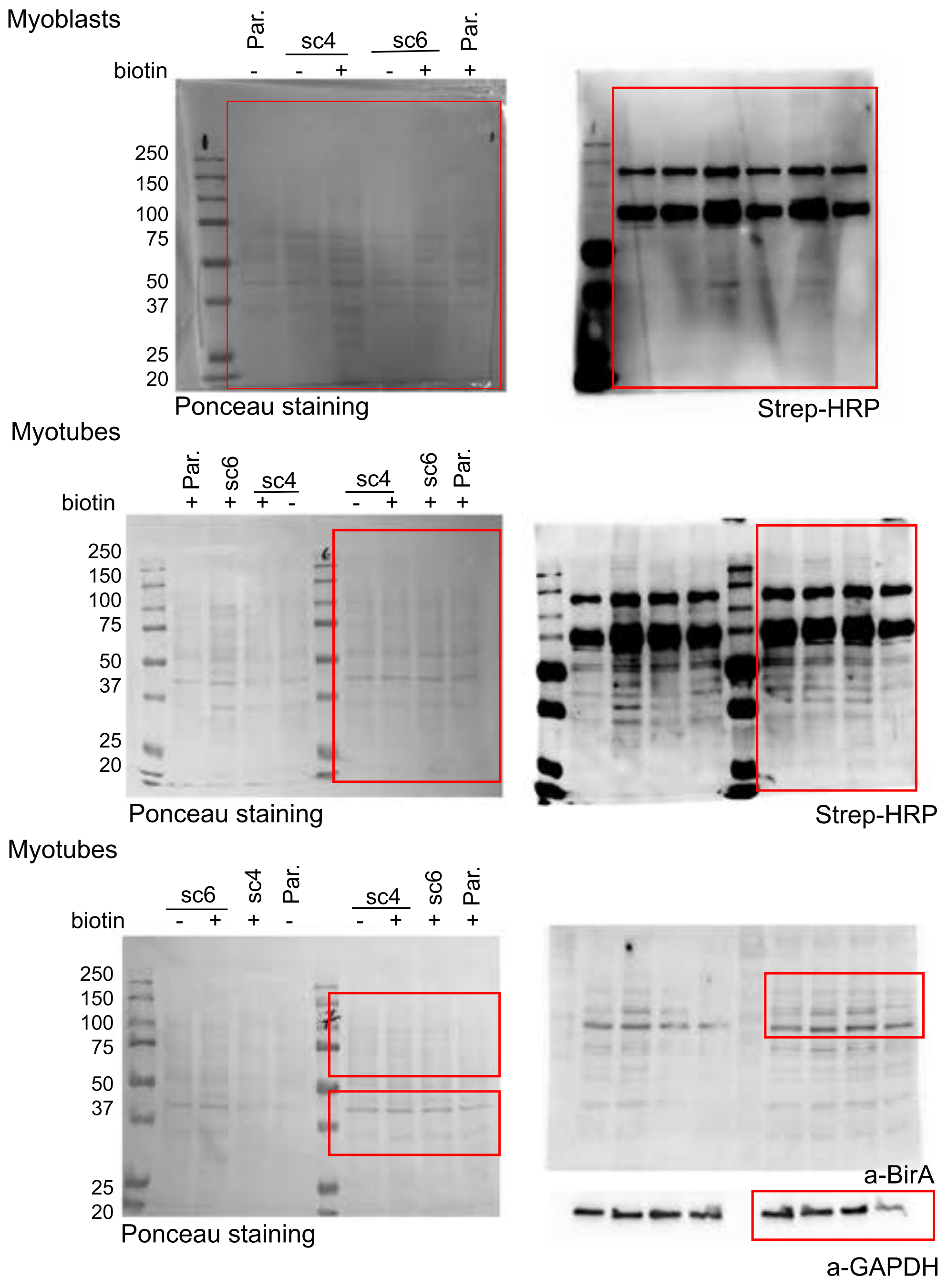

Supplementary Figure 11
Uncropped western blot for Figure 3A

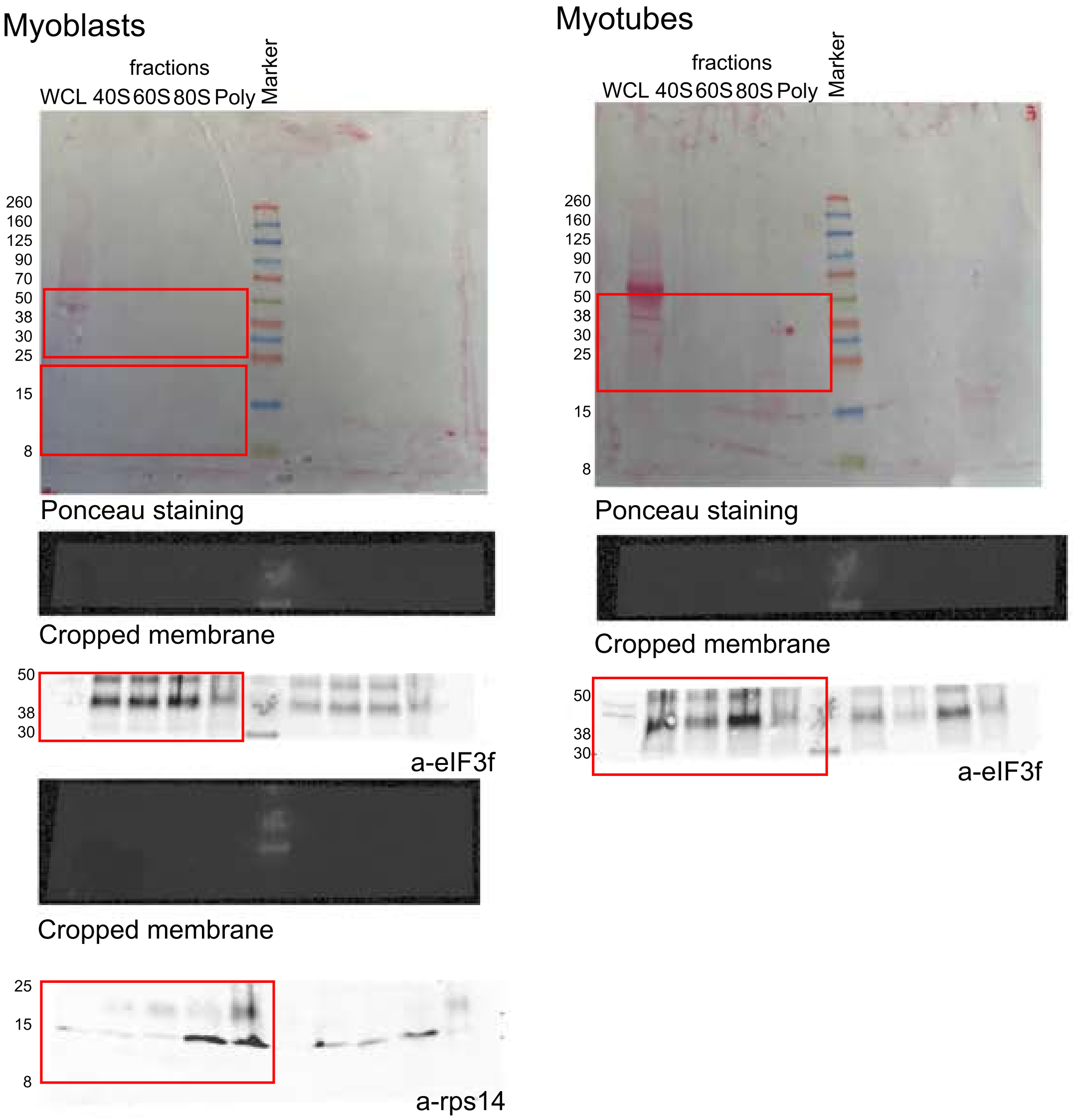

Supplementary Figure 12

Uncropped western blot for Figure 4B

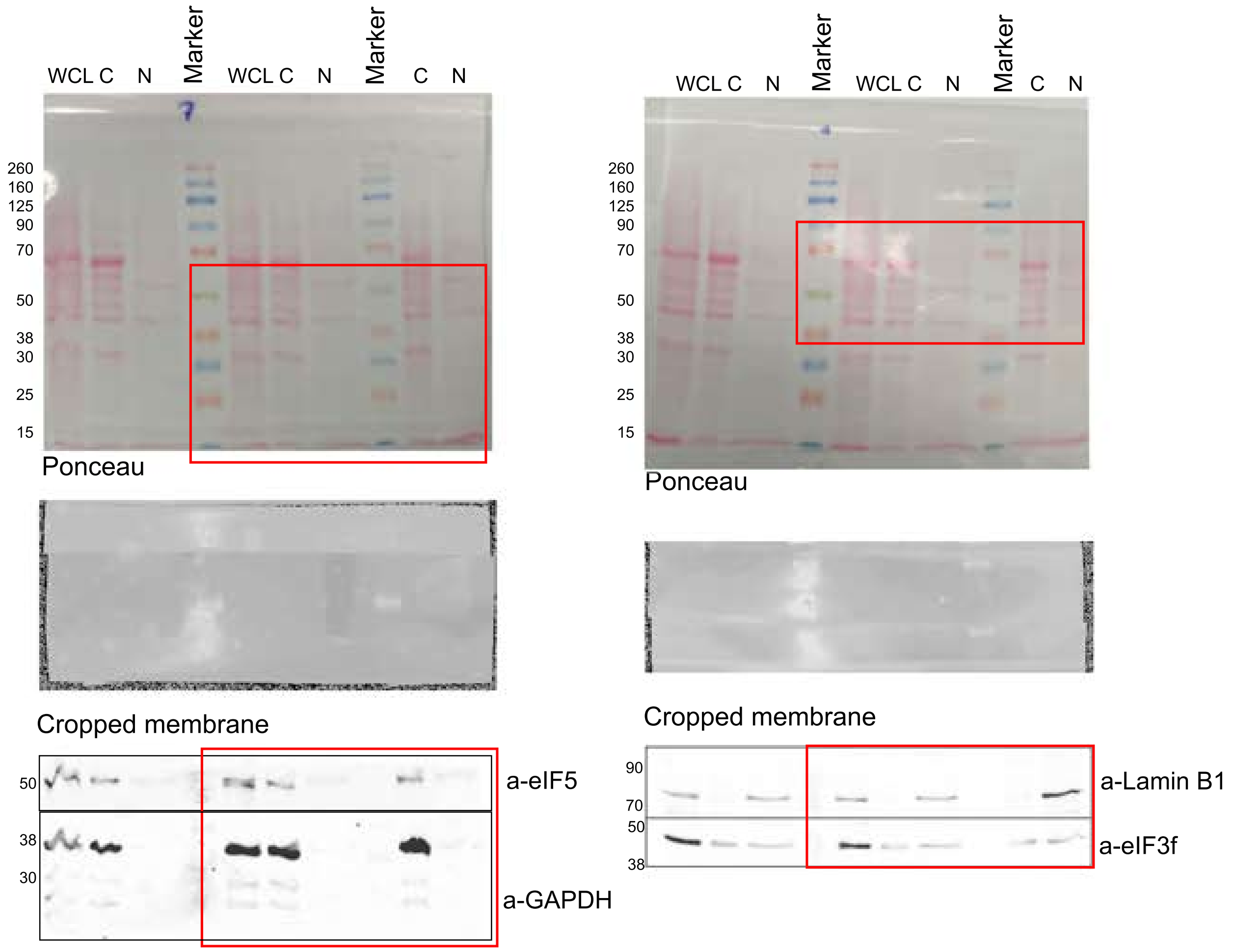

Supplementary Figure 13

Uncropped western blot for Figure 4D

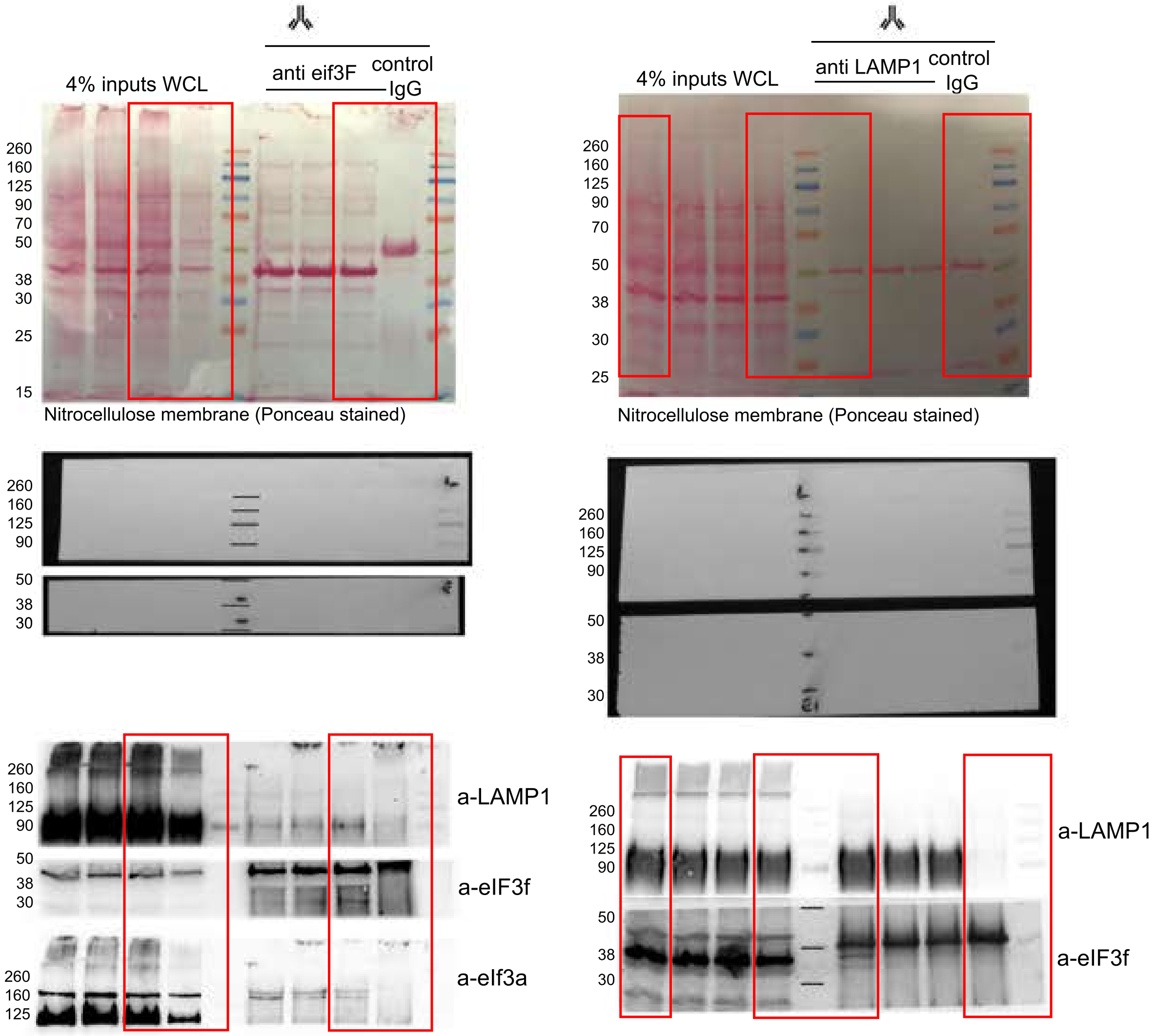

Supplementary Figure 14
Uncropped western blot for Figure 5B

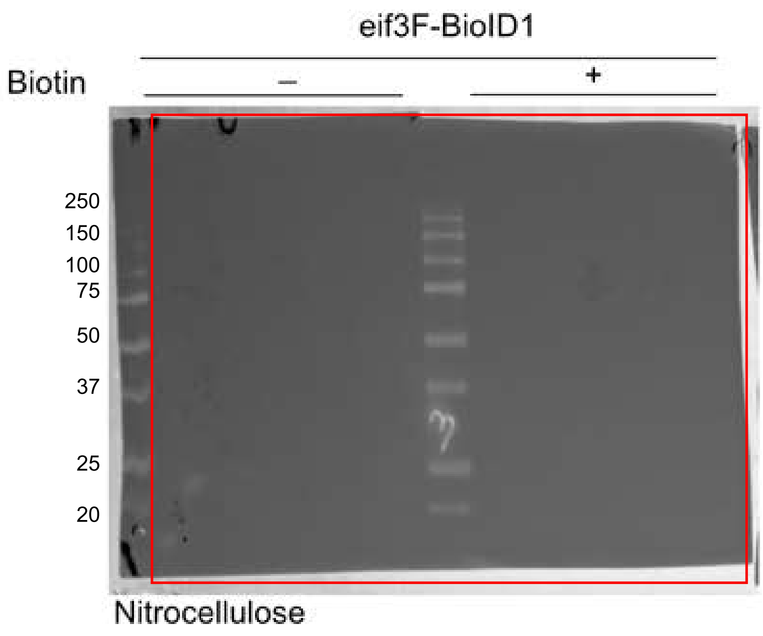

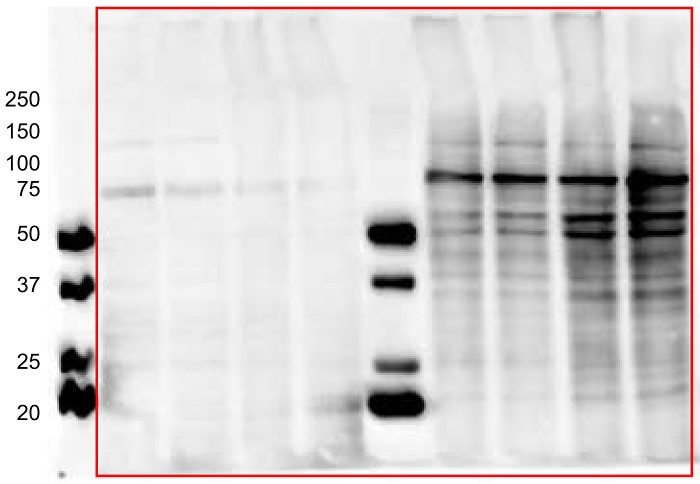

Supplementary Figure 15
Uncropped western blot for Supplementary Figure 1A

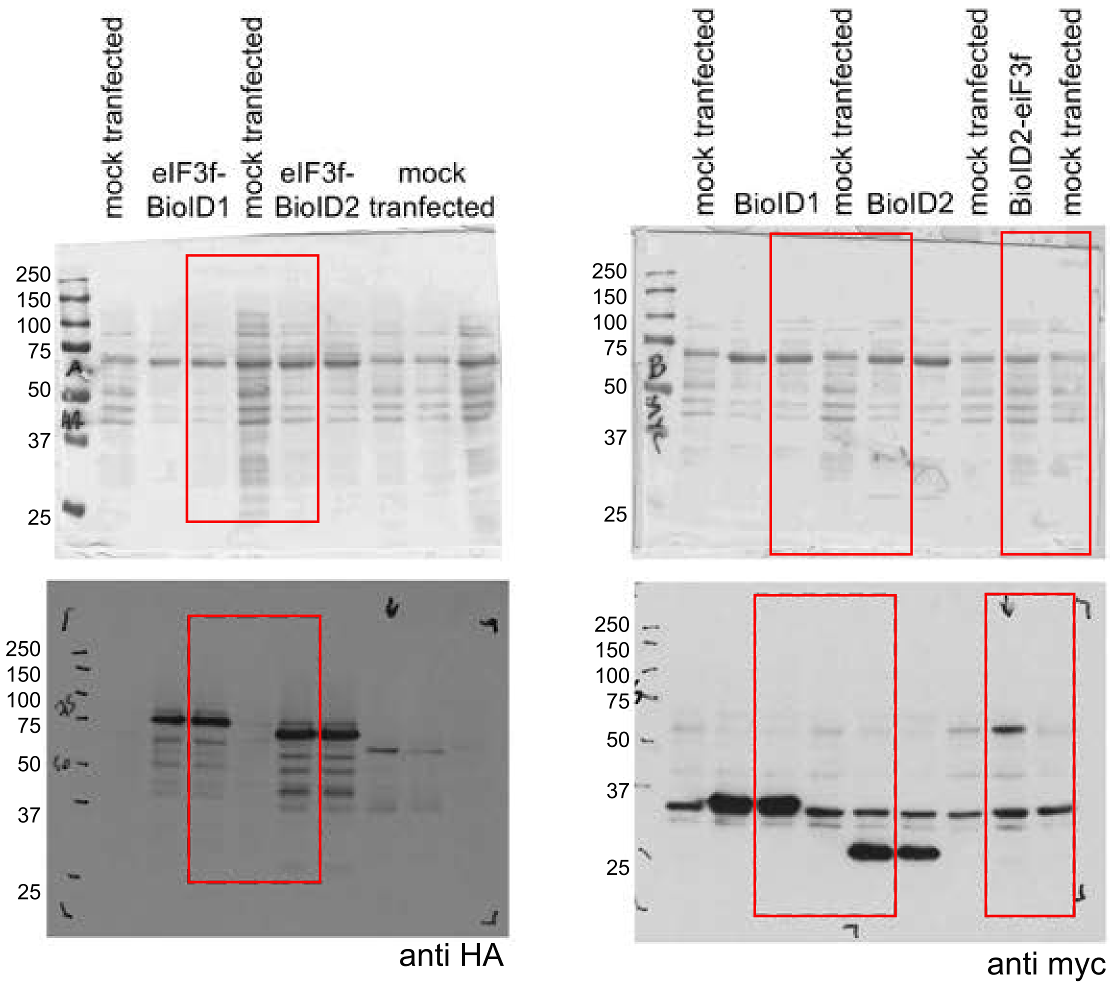

Supplementary Figure 16
Uncropped western blot for Supplementary Figure 2A

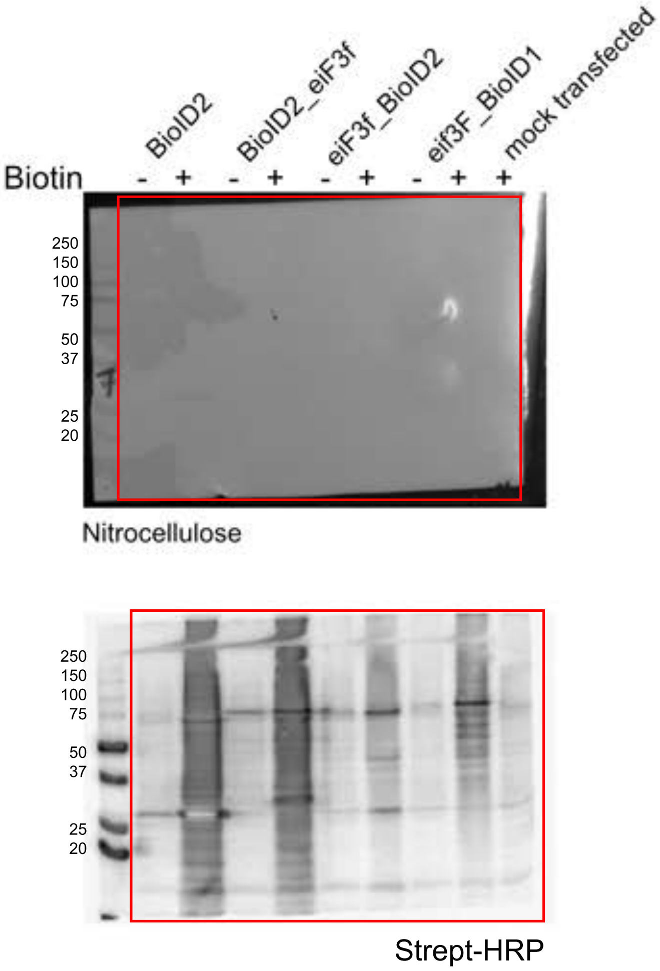

Supplementary Figure 17
Uncropped western blot for Supplementary Figure 2B

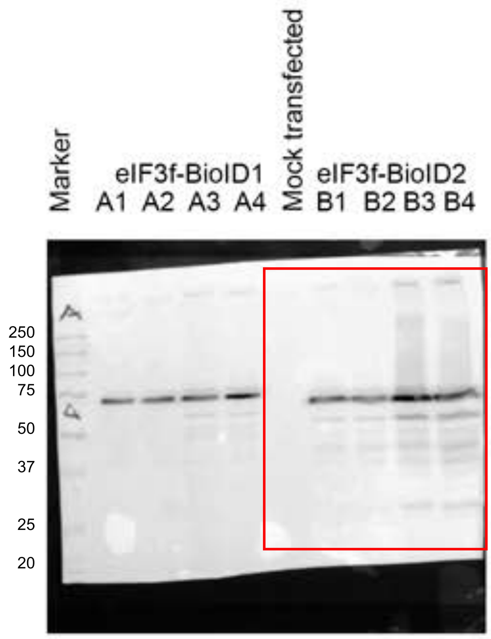

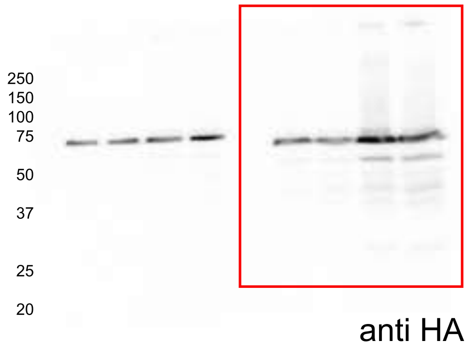

Supplementary Figure 18
Uncropped western blot for Supplementary Figure 2C

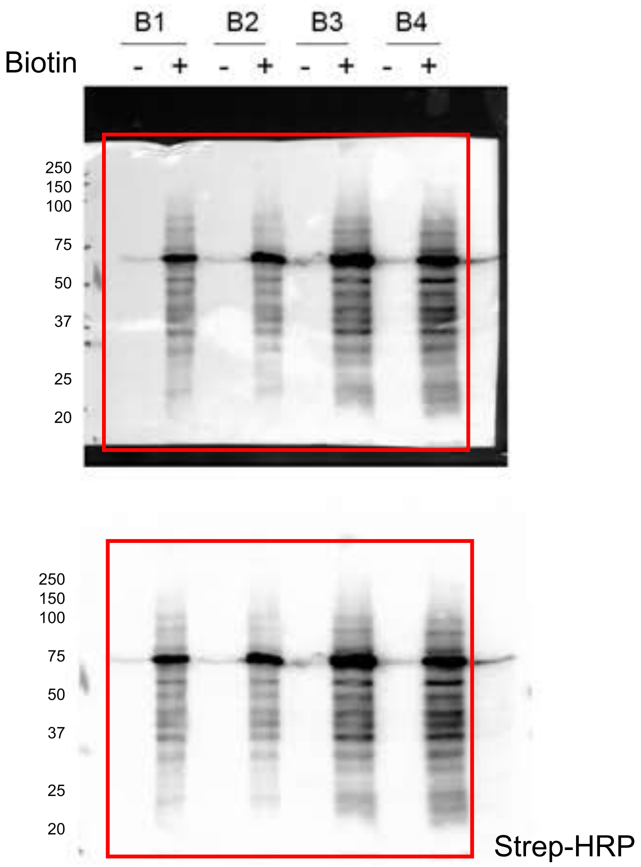

Supplementary Figure 19
Uncropped western blot for Supplementary Figure 2D

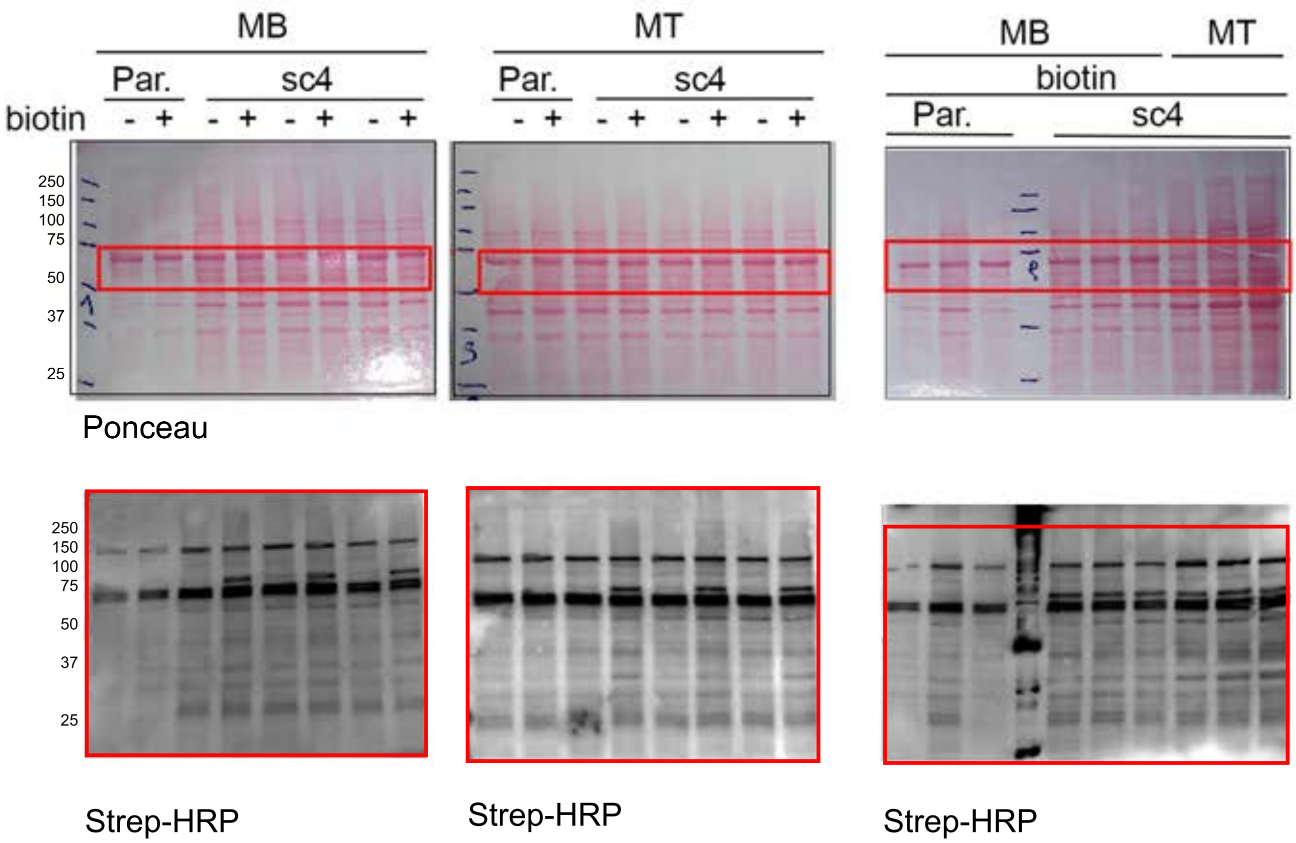

Supplementary Figure 20
Uncropped western blot for Supplementary Figure 4A

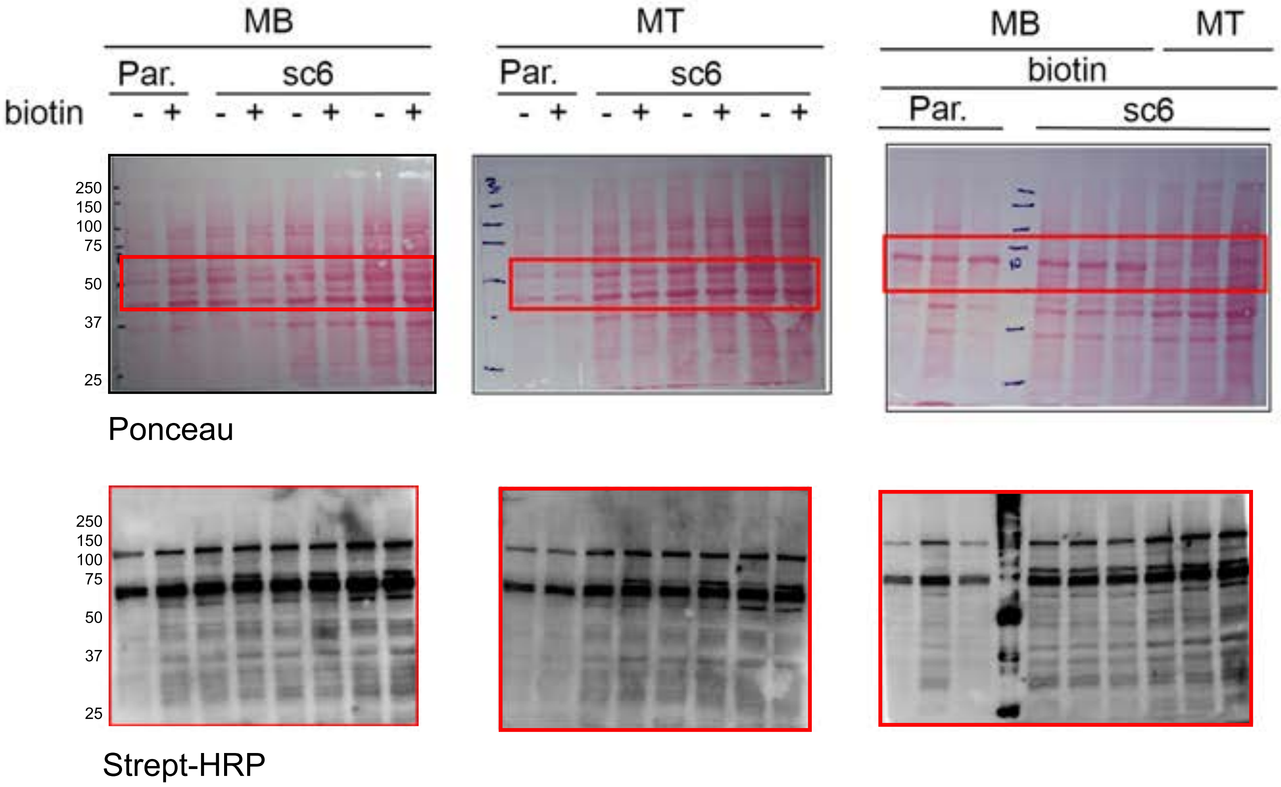

Supplementary Figure 21
Uncropped western blot for Supplementary Figure 4B

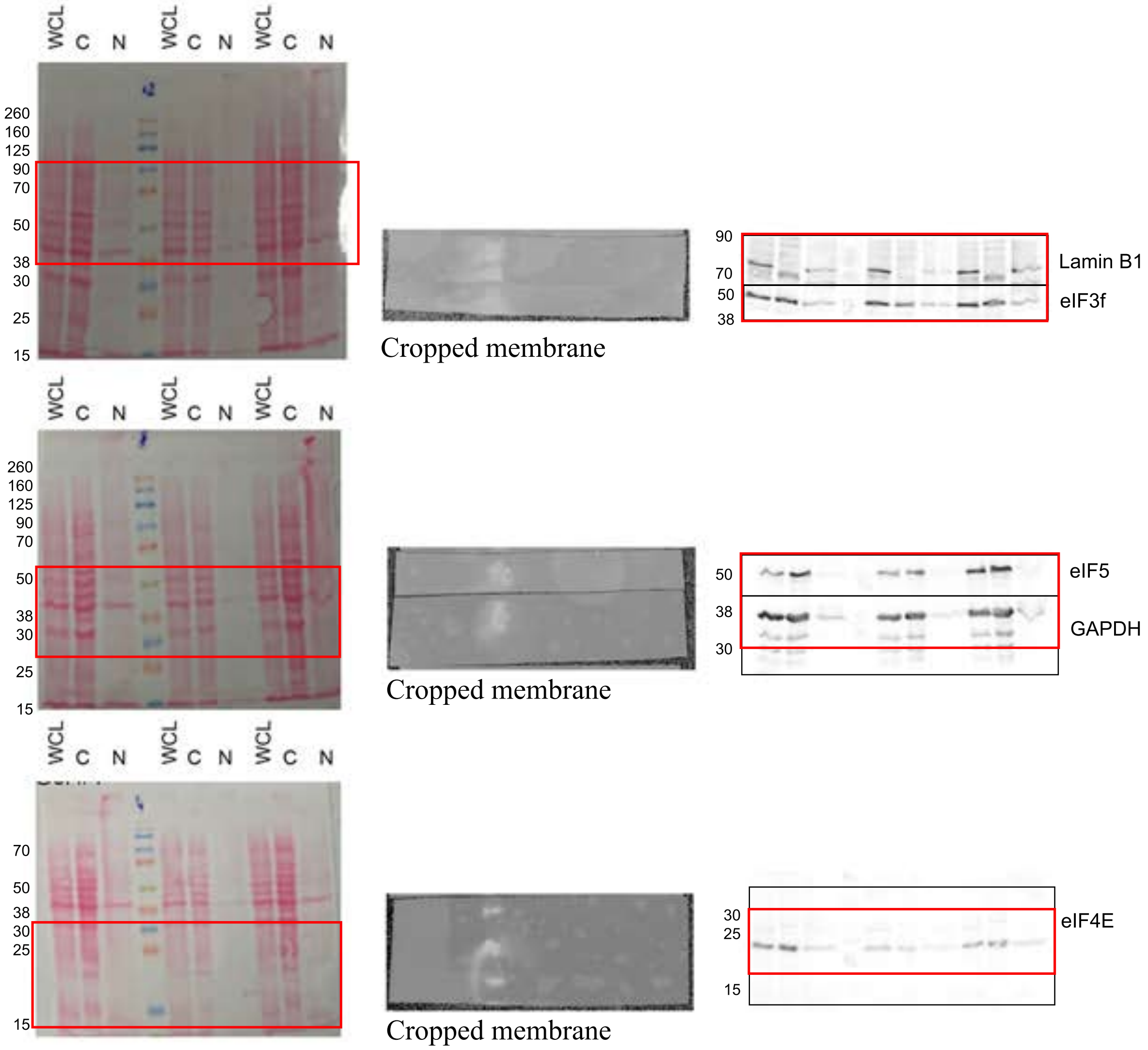

Supplementary Figure 22
Uncropped western blot for Supplementary Figure 6C

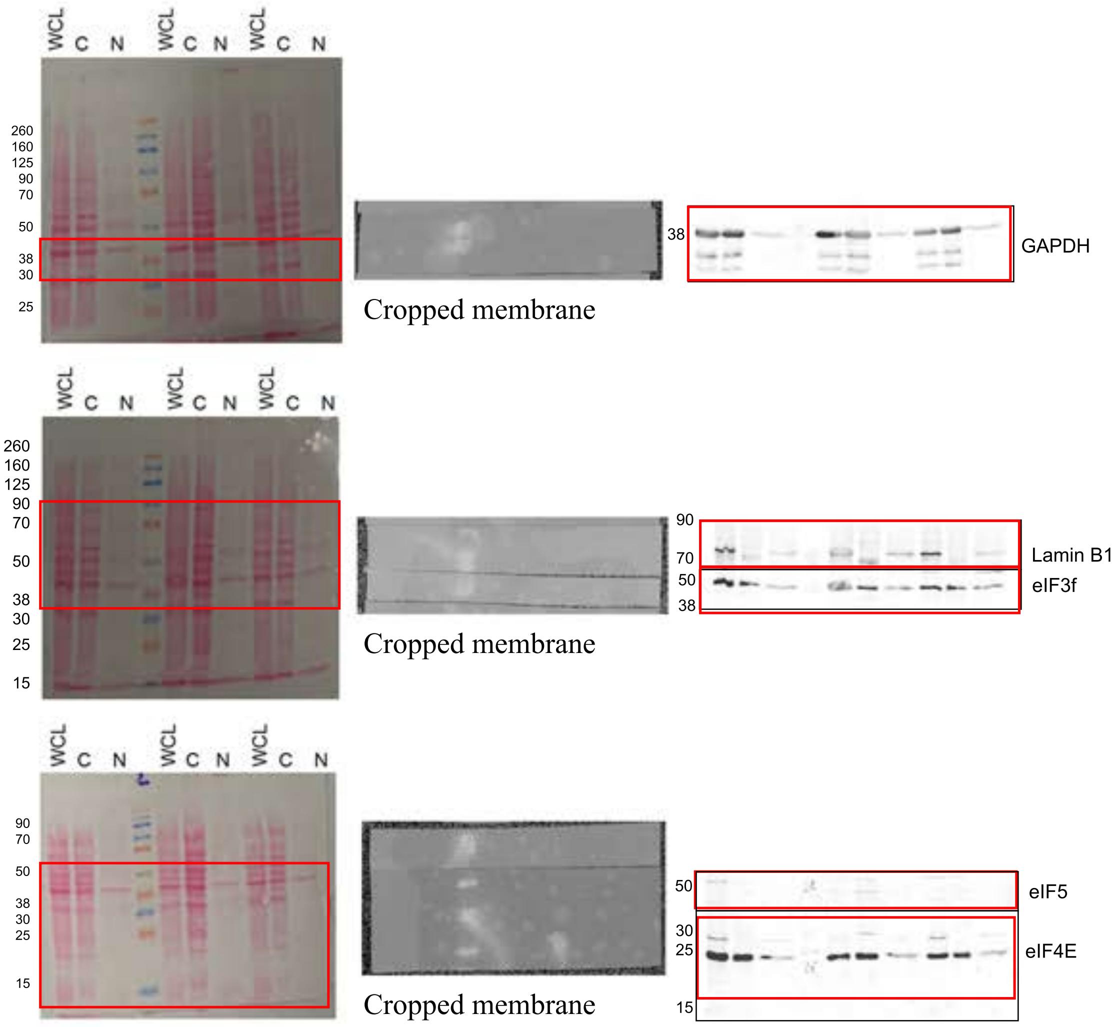

Supplementary Figure 23

Uncropped western blot for Supplementary Figure 6D

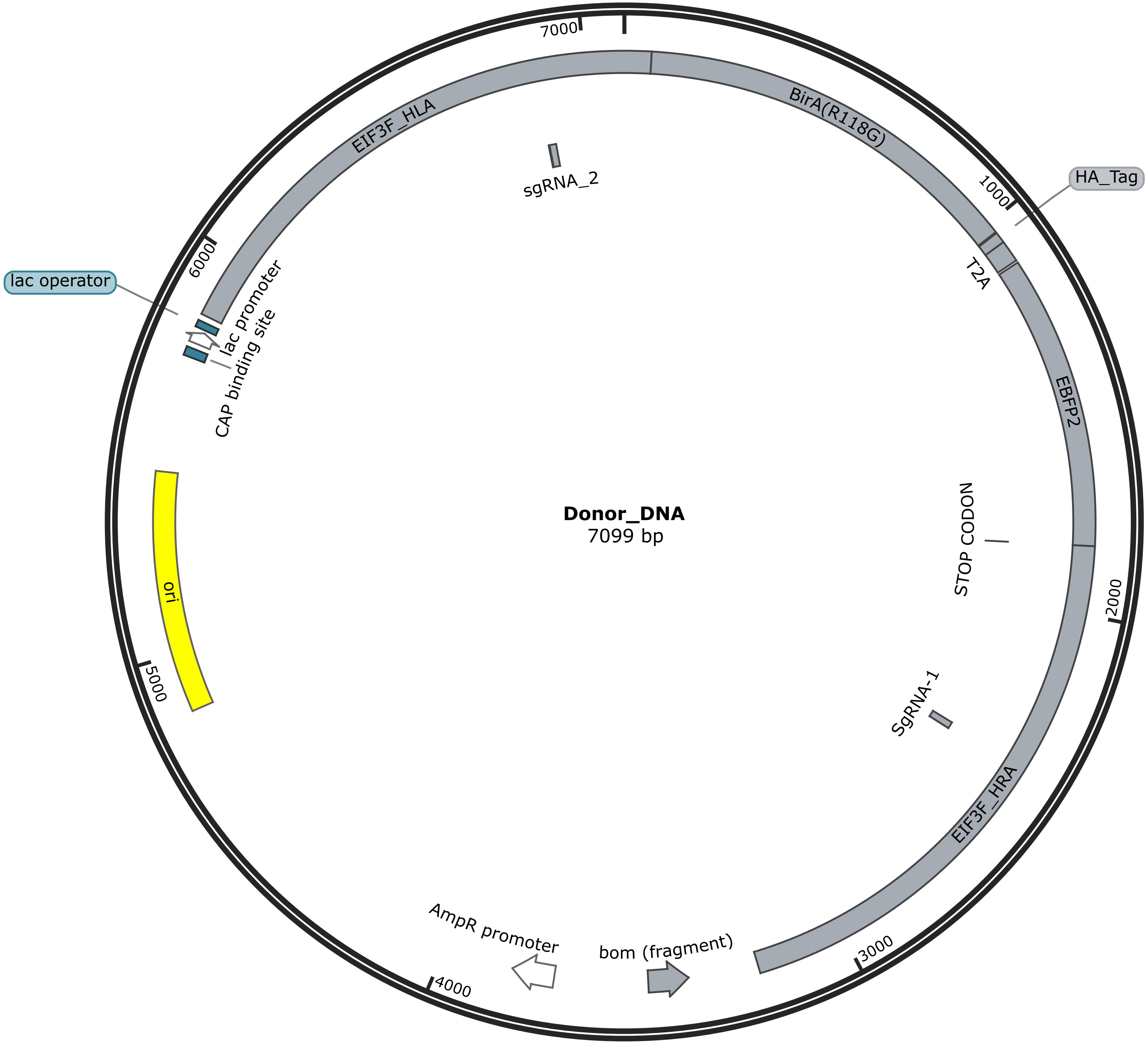
Created by SnapGene
Donor_DNA
7099 bp
7000
1000
2000
3000
4000
5000
6000
BirA(R118G)
HA_Tag
T2A
EBFP2
STOP CODON
SgRNA-1
EIF3F_HRA
bom (fragment)
AmpR promoter
ori
CAP binding site
lac promoter
lac operator
EIF3F_HLA
sgRNA_2

Donor_DNA_annotation_all_feature.dna

atggtgacctacctggccaacctcacacagtcacagattgcactcaatgaaaaacttgtaaacctgaaggacaacaccgt

gcccctgaagctgatcgccctgctggccaacggcgagttccactctggcgagcagctgggagagaccctgggaatgagca

gagccgccatcaacaagcacatccagacactgagagactggggagtggacgtgttcaccgtgcctggcaagggctacagc

ctgcctgagcctatccagctgctgaacgccaagcagatcctgggacagctggatggcggaagcgtggccgtgctgcctgt

gatcgactccaccaatcagtacctgctggacagaatcggagagctgaagtccggcgacgcctgcatcgccgagtaccagc

aggctggcagaggaggcagaggacggaagtggttcagcccattcggagccaacctgtacctgtccatgttctggagactg

gagcagggacctgctgctgccatcggactgagtctggtgatcggaatcgtgatggccgaggtgctgagaaagctgggagc

cgacaaggtgagagtgaagtggcctaatgacctgtacctccaggaccgcaagctggctggcatcctggtggagctgacag

gcaagacaggcgatgccgctcagatcgtgatcggagccggaatcaacatggccatgagaagagtggaggagagcgtggtg

aaccagggctggatcaccctgcaggaggctggcatcaacctggaccggaacaccctggccgccatgctgatcagagagct

gagagccgctctggagctgttcgagcaggagggactggctccttacctgagcagatgggagaagctggacaacttcatca

acagacctgtgaagctgatcatcggcgacaaggaaatcttcggcatctccagaggaatcgacaagcagggagctctgctg

ctggagcaggacggaatcatcaagccctggatgggcggagaaatctccctgagaagcgcagagaaggcttacccttacga

tgtaccggattacgcagagggcagaggaagtctgctaacatgcggtgacgtggaggagaatcccggccctgctagcatgg

tgagcaagggcgaggagctgttcaccggggtggtgcccatcctggtcgagctggacggcgacgtaaacggccacaagttc

agcgtgaggggcgagggcgagggcgatgccaccaacggcaagctgaccctgaagttcatctgcaccaccggcaagctgcc

cgtgccctggcccaccctcgtgaccaccctgagccacggcgtgcagtgcttcgcccgctaccccgaccacatgaagcagc

acgacttcttcaagtccgccatgcccgaaggctacgtccaggagcgcaccatcttcttcaaggacgacggcacctacaag

acccgcgccgaggtgaagttcgagggcgacaccctggtgaaccgcatcgagctgaagggcgtcgacttcaaggaggacgg

caacatcctggggcacaagctggagtacaacttcaacagccacaacatctatatcatggccgtcaagcagaagaacggca

tcaaggtgaacttcaagatccgccacaacgtggaggacggcagcgtgcagctcgccgaccactaccagcagaacacccccc

atcggcgacggccccgtgctgctgcccgacagccactacctgagcacccagtccgtgctgagcaaagaccccaacgagaa

gcgcgatcacatggtcctgctggagttccgcaccgccgccgggatcactctcggcatggacgagctgtacaagtgaatgg

accccaagcagtacacttgctggtctaggtattaaccccaggactcagaagtgaaggagaaatgggtttttttgtggtctt

gagtcacactgagatagtcagttgtgtgtgactctaataaacggagcctaccttttgtaaattaatttcatcttatgtga

gtttattgccgggtggaagggaggaaaatgttttagatcacacagaaactaggaagtgaatattgtaggtagagaagttg

gttatgtgtttggcatgttactgttttaccaaactgttcttttggttttcaatatggaaaggtgtccattggcaaaacaa

ccttttcttgaggattcatgtgatttctacaggaaatgaaaataagaatccatggagaaaaaatagctaagaaaattgga

tgcttaatctgaactaaaagagtagagtgtgtagtcactatcttaaaatacattaatagtaagatggagctctttgccac

tttggttcggggaggcagaattaagatctgtgggttaaagattaaagaggtttccccaaaacttcagaaaattcttaggt

gctatctgtaactcgagtatagagccttgagttatactgaggtctgccagtggctcaatttaagcaaagactagatggca

tttcccagaggccttgtaggctagagtacttgcctgtgtaacttcagacatccatttcctctggtatcctggagttccgc

ctcgccactgtgtgtcttagaaataagtcattattcaagaactcaggaaattatacgttggagtgaaaaggtgagtgaag

gcaatggggaatcaaaaaggcctggatcctagttctgaggtatgtgtagccttaggaaagataaattataagagtcataa
gatatggatcataaacatacatagtccattagggtattatgaggattccatgaattaataaatgtcaggttaatcaacac
ttgtggagaatatcacctggtatgtagtaagcacagataagtggtagatactatgatgcctaaaggtgaatagtttgaaa
gtaataagcaacatgaaccccagcctggaactcagttctgagatatgtacctcaggaaatataagttatgggagtcataa
agtgtggatagtaatgatagataatttcttacaggtagggaaatgtaaacagatcttatcggcaaaatgataatgacagg
ccaaattgttaaaggtaggttaaaagtagacagcatggtgggaaggttaatcagagtcaaaatgtttcagtgttcttaca
tttttgggagtagccagttaatatcaacttagtctttttaagtatgcatgataaaattccgaggttaattgctagaagta
tatataatctctcaatgaacgagctcgaattcactggccgtcgttttacaacgtcgtgactgggaaaaccctggcgttac
ccaacttaatcgccttgcagcacatccccctttcgccagctggcgtaatagcgaagaggcccgcaccgatcgcccttccc
aacagttgcgcagcctgaatggcgaatggcgcctgatgcggtattttctccttacgcatctgtgcggtatttcacaccgc
atatggtgcactctcagtacaatctgctctgatgccgcatagttaagccagccccgacacccgccaacacccgctgacgc
gccctgacgggcttgtctgctcccggcatccgcttacagacaagctgtgaccgtctccgggagctgcatgtgtcagaggt
tttcaccgtcatcaccgaaacgcgcgagacgaaagggcctcgtgatacgcctatttttataggttaatgtcatgataata
atggtttcttagacgtcaggtggcacttttcggggaaatgtgcgcggaacccctatttgtttatttttctaaatacattc
aaatatgtatccgctcatgagacaataaccctgataaatgcttcaataatattgaaaaaggaagagtatgagtattcaac
atttccgtgtcgcccttattcccttttttgcggcattttgccttcctgtttttgctcacccagaaacgctggtgaaagta
aaagatgctgaagatcagttgggtgcacgagtgggttacatcgaactggatctcaacagcggtaagatccttgagagttt
tcgccccgaagaacgttttccaatgatgagcacttttaaagttctgctatgtggcgcggtattatcccgtattgacgccg
ggcaagagcaactcggtcgccgcatacactattctcagaatgacttggttgagtactcaccagtcacagaaaagcatctt
acggatggcatgacagtaagagaattatgcagtgctgccataaccatgagtgataacactgcggccaacttacttctgac
aacgatcggaggaccgaaggagctaaccgcttttttgcacaacatgggggatcatgtaactcgccttgatcgttgggaac
cggagctgaatgaagccataccaaacgacgagcgtgacaccacgatgcctgtagcaatggcaacaacgttgcgcaaacta
ttaactggcgaactacttactctagcttcccggcaacaattaatagactggatggaggcggataaagttgcaggaccact
tctgcgctcggcccttccggctggctggtttattgctgataaatctggagccggtgagcgtgggtctcgcggtatcattg
cagcactggggccagatggtaagccctcccgtatcgtagttatctacacgacggggagtcaggcaactatggatgaacga
aatagacagatcgctgagataggtgcctcactgattaagcattggtaactgtcagaccaagtttactcatatatacttta
gattgatttaaaacttcatttttaatttaaaaggatctaggtgaagatcctttttgataatctcatgaccaaaatccctt
aacgtgagttttcgttccactgagcgtcagaccccgtagaaaagatcaaaggatcttcttgagatcctttttttctgcgc
gtaatctgctgcttgcaaacaaaaaaaccaccgctaccagcggtggtttgtttgccggatcaagagctaccaactctttt
tccgaaggtaactggcttcagcagagcgcagataccaaatactgttcttctagtgtagccgtagttaggccaccacttca
agaactctgtagcaccgcctacatacctcgctctgctaatcctgttaccagtggctgctgccagtggcgataagtcgtgt
cttaccgggttggactcaagacgatagttaccggataaggcgcagcggtcgggctgaacggggggttcgtgcacacagcc
cagcttggagcgaacgacctacaccgaactgagatacctacagcgtgagctatgagaaagcgccacgcttcccgaaggga
gaaaggcggacaggtatccggtaagcggcagggtcggaacaggagagcgcacgagggagcttccagggggaaacgcctgg

tatctttatagtcctgtcgggtttcgccacctctgacttgagcgtcgatttttgtgatgctcgtcaggggggcggagcct

atggaaaaacgccagcaacgcggcctttttacggttcctggccttttgctggccttttgctcacatgttctttcctgcgt

tatcccctgattctgtggataaccgtattaccgcctttgagtgagctgataccgctcgccgcagccgaacgaccgagcgc

agcgagtcagtgagcgaggaagcggaagagcgcccaatacgcaaaccgcctctccccgcgcgttggccgattcattaatg

cagctggcacgacaggtttcccgactggaaagcgggcagtgagcgcaacgcaattaatgtgagttagctcactcattagg

caccccaggctttacactttatgcttccggctcgtatgttgtgtggaattgtgagcggataacaatttcacacaggaaac

agctatgaccatgattacgccaatgatcagcaccagaggccacataccattcacatgagatcaaaaccggtgatgagcat

accaggtaaaaggagttgagctctattaggaggaagaaaagctgataagggcacagcaggaagtgtgactgagcagactg

agtctaaggggagttgggaaacagagttagtaggacgttaagaccatctctttcctggtgggatgactgaattctctctc

ttactgcccaccactgccgctgccaccctgccaaccaacttccatagttgacctgatcatgaagacctgctttagcccca

acagagtgattggactctcaagtgacttgcagcaagtaggaggggcatcagctcgcatccaggatgccctgagtacagtg

ttgcaatatgcagaggatgtactggtgagaggggaaagaaaaaacaaaggggaggacatagttctctatcctgggatca

ctgaggcatgtgctgatgaaatggtagggctcagtggttataattaactgcctcatcgagtctttgttttggtactcagt

ctggaaaggtgtcagctgacaatactgtgggccgcttcctgatgagcctggttaaccaagtaccgaaaatagttcccgat

gactttgagaccatgctcaacagcaacatcaatgtgagtgcccttcctgagccccttcttgcctggtttcttcccccacc

tcagcacatacactcaaatgtgaaaagagttgggtgaggtgacctggagtaggatagagacagggcttcacctatagccc

cactgaagtccaggttgggaggaaggaagaggtgtgttatgcgtggttggagacttccttccttctcccatgattccatt

gtctcatttactgctgatactactgtgacttactgtctaaattcgtgatggaaagaaacactaaaattcagaggttaatg

aaaatgcaattacttatttcccccatctgagttcacagacccccctatattccatccatgggtttcagctaaggcacaact

agtctataagtgtatcagtagagataagaaagccttctcaagcatttcctttctgtcttctaccttttttgacttttattc

agtctctcctagctgtcatttatccacagctgtcctcatacccagtgtctaccatagagctggccaaagccagccttttt

gctccctttccacccaaccccccactcattgtgtattctttgtcttccaggaccttttg

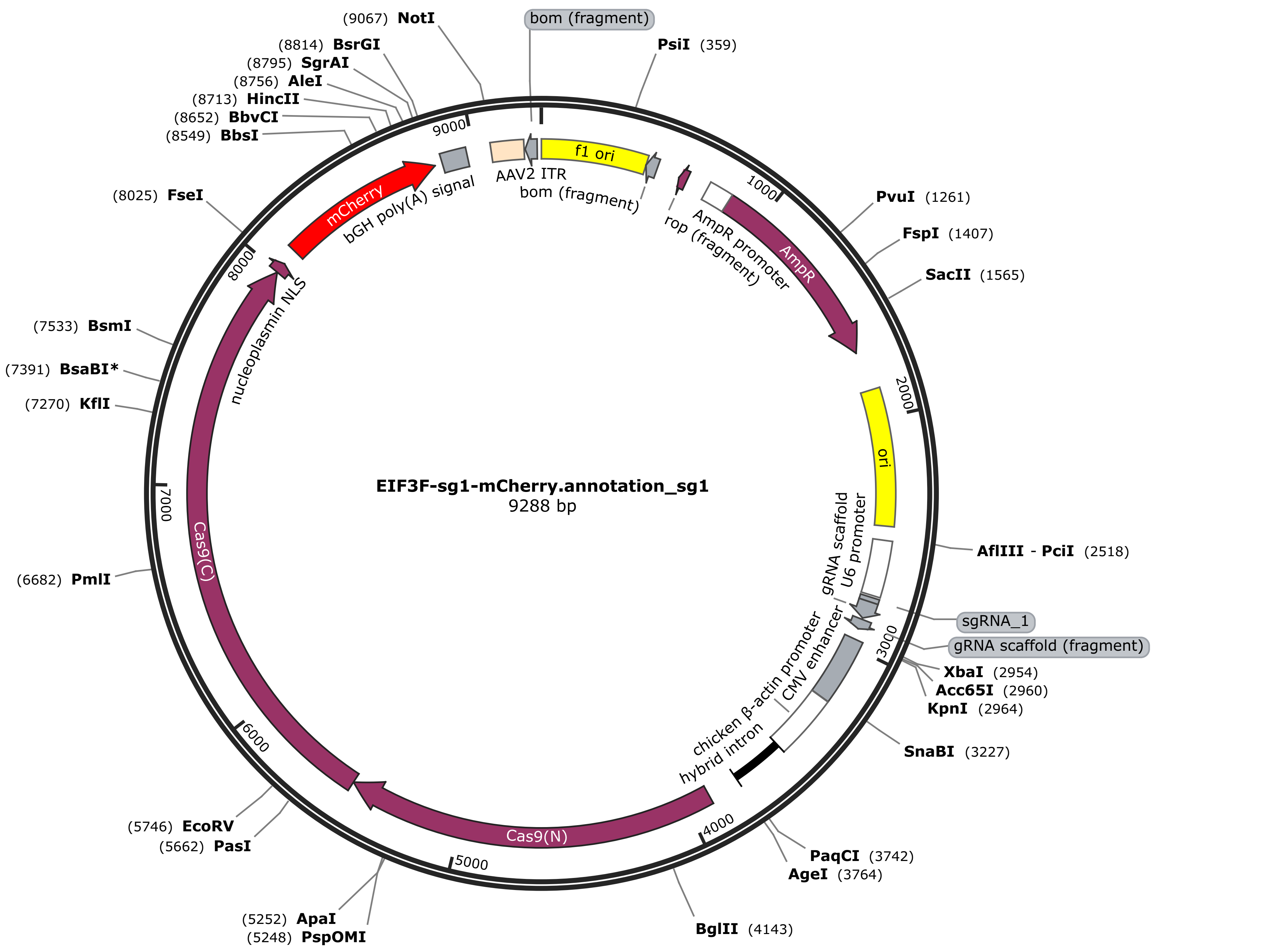
Created by SnapGene
EIF3F-sg1-mCherry.annotation_sg1
9288 bp
(9067) NotI
bom (fragment)
PsiI (359)
(8814) BsrGI
(8795) SgrAI
(8756) AleI
(8713) HincII
(8652) BbvCI
(8549) BbsI
(8025) FseI
PvuI (1261)
FspI (1407)
SacII (1565)
(7533) BsmI
(7391) BsaBI*
(7270) KflI
(6682) PmlI
AflIII - PciI (2518)
sgRNA_1
gRNA scaffold (fragment)
XbaI (2954)
Acc65I (2960)
KpnI (2964)
SnaBI (3227)
(5746) EcoRV
(5662) PasI
PaqCI (3742)
AgeI (3764)
(5252) ApaI
(5248) PspOMI
BglII (4143)
mCherry
bGH poly(A) signal
AAV2 ITR
f1 ori
bom (fragment)
rop (fragment)
AmpR promoter
AmpR
ori
gRNA scaffold
U6 promoter
CMV enhancer
chicken β-actin promoter
hybrid intron
Cas9(N)
Cas9(C)
nucleoplasmin NLS
1000
2000
3000
4000
5000
6000
7000
8000
9000

EIF3F-sg1-mCherry.annotation_sg1.dna

acgcgccctgtagcggcgcattaagcgcggcgggtgtggtggttacgcgcagcgtgaccgctacacttgccagcgccta
gcgcccgctcctttcgctttcttcccttcctttctcgccacgttcgccggctttccccgtcaagctctaaatcgggggct
ccctttagggttccgatttagtgctttacggcacctcgaccccaaaaaacttgatttgggtgatggttcacgtagtgggc
catcgccctgatagacggtttttcgccctttgacgttggagtccacgttctttaatagtggactcttgttccaaactgga
acaacactcaactctatctcgggctattcttttgatttataagggattttgccgatttcggtctattggttaaaaaatga
gctgatttaacaaaaatttaacgcgaattttaacaaaatattaacgtttacaattttatggtgcactctcagtacaatct
gctctgatgccgcatagttaagccagccccgacacccgccaacacccgctgacgcgccctgacgggcttgtctgctcccg
gcatccgcttacagacaagctgtgaccgtctccgggagctgcatgtgtcagaggttttcaccgtcatcaccgaaacgcgc
gagacgaaagggcctcgtgatacgcctatttttataggttaatgtcatgataataatggtttcttagacgtcaggtggca
cttttcggggaaatgtgcgcggaacccctatttgtttatttttctaaatacattcaaatatgtatccgctcatgagacaa
taaccctgataaatgcttcaataatattgaaaaaggaagagtatgagtattcaacatttccgtgtcgcccttattccctt
ttttgcggcattttgccttcctgtttttgctcacccagaaacgctggtgaaagtaaaagatgctgaagatcagttgggtg
cacgagtgggttacatcgaactggatctcaacagcggtaagatccttgagagttttcgccccgaagaacgttttccaatg
atgagcacttttaaagttctgctatgtggcgcggtattatcccgtattgacgccgggcaagagcaactcggtcgccgcat
acactattctcagaatgacttggttgagtactcaccagtcacagaaaagcatcttacggatggcatgacagtaagagaat
tatgcagtgctgccataaccatgagtgataacactgcggccaacttacttctgacaacgatcggaggaccgaaggagcta
accgcttttttgcacaacatgggggatcatgtaactcgccttgatcgttgggaaccggagctgaatgaagccataccaaa
cgacgagcgtgacaccacgatgcctgtagcaatggcaacaacgttgcgcaaactattaactggcgaactacttactctag
cttcccggcaacaattaatagactggatggaggcggataaagttgcaggaccacttctgcgctcggcccttccggctggc
tggtttattgctgataaatctggagccggtgagcgtggaagccgcggtatcattgcagcactggggccagatggtaagcc
ctcccgtatcgtagttatctacacgacggggagtcaggcaactatggatgaacgaaatagacagatcgctgagataggtg
cctcactgattaagcattggtaactgtcagaccaagtttactcatatatactttagattgatttaaaacttcatttttaa
tttaaaaggatctaggtgaagatcctttttgataatctcatgaccaaaatcccttaacgtgagttttcgttccactgagc
gtcagaccccgtagaaaagatcaaaggatcttcttgagatcctttttttctgcgcgtaatctgctgcttgcaaacaaaaa
aaccaccgctaccagcggtggtttgtttgccggatcaagagctaccaactctttttccgaaggtaactggcttcagcaga
gcgcagataccaaatactgttcttctagtgtagccgtagttaggccaccacttcaagaactctgtagcaccgcctacata
cctcgctctgctaatcctgttaccagtggctgctgccagtggcgataagtcgtgtcttaccgggttggactcaagacgat
agttaccggataaggcgcagcggtcgggctgaacggggggttcgtgcacacagcccagcttggagcgaacgacctacacc
gaactgagatacctacagcgtgagctatgagaaagcgccacgcttcccgaagggagaaaggcggacaggtatccggtaag
cggcagggtcggaacaggagagcgcacgagggagcttccagggggaaacgcctggtatctttatagtcctgtcgggtttc
gccacctctgacttgagcgtcgatttttgtgatgctcgtcaggggggcggagcctatggaaaaacgccagcaacgcggcc
tttttacggttcctggccttttgctggccttttgctcacatgtgagggcctatttcccatgattccttcatatttgcata
tacgatacaaggctgttagagagataattggaattaatttgactgtaaacacaaagatattagtacaaaatacgtgacgt

agaaagtaataatttcttgggtagtttgcagttttaaaattatgttttaaaatggactatcatatgcttaccgtaacttg
aaagtatttcgatttcttggctttatatatcttgtggaaaggacgaaacaccgtaggtgctatctgtaactcggttttag
agctagaaatagcaagttaaaataaggctagtccgttatcaacttgaaaaagtggcaccgagtcggtgctttttttgtttt
agagctagaaatagcaagttaaaataaggctagtccgtttttagcgcgtgcgccaattctgcagacaaatggctctagag
gtacccgttacataacttacggtaaatggcccgcctggctgaccgcccaacgacccccgcccattgacgtcaatagtaac
gccaatagggactttccattgacgtcaatgggtggagtatttacggtaaactgcccacttggcagtacatcaagtgtatc
atatgccaagtacgccccctattgacgtcaatgacggtaaatggcccgcctggcattgtgcccagtacatgaccttatgg
gactttcctacttggcagtacatctacgtattagtcatcgctattaccatggtcgaggtgagccccacgttctgcttcac
tctccccatctcccccccctccccacccccaattttgtatttatttattttttaattattttgtgcagcgatgggggcgg
ggggggggggggggcgcgcgccaggcggggcggggcggggcgaggggcggggcggggcgaggcggagaggtgcggcggca
gccaatcagagcggcgcgctccgaaagtttccttttatggcgaggcggcggcggcggcggccctataaaaagcgaagcgc
gcggcgggcgggagtcgctgcgcgctgccttcgccccgtgccccgctccgccgccgcctcgcgccgcccgccccggctct
gactgaccgcgttactcccacaggtgagcgggcgggacggcccttctcctccgggctgtaattagctgagcaagaggtaa
gggtttaagggatggttggttggtggggtattaatgtttaattacctggagcacctgcctgaaatcactttttttcaggt
tggaccggtgccaccatggactataaggaccacgacggagactacaaggatcatgatattgattacaaagacgatgacga
taagatggccccaaagaagaagcggaaggtcggtatccacggagtcccagcagccgacaagaagtacagcatcggcctgg
acatcggcaccaactctgtgggctgggccgtgatcaccgacgagtacaaggtgcccagcaagaaattcaaggtgctgggc
aacaccgaccggcacagcatcaagaagaacctgatcggagccctgctgttcgacagcggcgaaacagccgaggccacccg
gctgaagagaaccgccagaagaagatacaccagacggaagaaccggatctgctatctgcaagagatcttcagcaacgaga
tggccaaggtggacgacagcttcttccacagactggaagagtccttcctggtggaagaggataagaagcacgagcggcac
cccatcttcggcaacatcgtggacgaggtggcctaccacgagaagtaccccaccatctaccacctgagaaagaaactggt
ggacagcaccgacaaggccgacctgcggctgatctatctggccctggcccacatgatcaagttccggggccacttcctga
tcgagggcgacctgaaccccgacaacagcgacgtggacaagctgttcatccagctggtgcagacctacaaccagctgttc
gaggaaaaccccatcaacgccagcggcgtggacgccaaggccatcctgtctgccagactgagcaagagcagacggctgga
aaatctgatcgcccagctgcccggcgagaagaagaatggcctgttcggaaacctgattgccctgagcctgggcctgaccc
ccaacttcaagagcaacttcgacctggccgaggatgccaaactgcagctgagcaaggacacctacgacgacgacctggac
aacctgctggcccagatcggcgaccagtacgccgacctgtttctggccgccaagaacctgtccgacgccatcctgctgag
cgacatcctgagagtgaacaccgagatcaccaaggcccccctgagcgcctctatgatcaagagatacgacgagcaccacc
aggacctgaccctgctgaaagctctcgtgcggcagcagctgcctgagaagtacaaagagattttcttcgaccagagcaag
aacggctacgccggctacattgacggcggagccagccaggaagagttctacaagttcatcaagcccatcctggaaaagat
ggacggcaccgaggaactgctcgtgaagctgaacagagaggacctgctgcggaagcagcggaccttcgacaacggcagca
tcccccaccagatccacctgggagagctgcacgccattctgcggcggcaggaagatttttacccattcctgaaggacaac
cgggaaaagatcgagaagatcctgaccttccgcatcccctactacgtgggccctctggccaggggaaacagcagattcgc
ctggatgaccagaaagagcgaggaaaccatcaccccctggaacttcgaggaagtggtggacaagggcgcttccgcccaga

gcttcatcgagcggatgaccaacttcgataagaacctgcccaacgagaaggtgctgcccaagcacagcctgctgtacgag
tacttcaccgtgtataacgagctgaccaaagtgaaatacgtgaccgagggaatgagaaagcccgccttcctgagcggcga
gcagaaaaaggccatcgtggacctgctgttcaagaccaaccggaaagtgaccgtgaagcagctgaaagaggactacttca
agaaaatcgagtgcttcgactccgtggaaatctccggcgtggaagatcggttcaacgcctccctgggcacataccacgat
ctgctgaaaattatcaaggacaaggacttcctggacaatgaggaaaacgaggacattctggaagatatcgtgctgaccct
gacactgtttgaggacagagagatgatcgaggaacggctgaaaacctatgcccacctgttcgacgacaaagtgatgaagc
agctgaagcggcggagatacaccggctggggcaggctgagccggaagctgatcaacggcatccgggacaagcagtccggc
aagacaatcctggatttcctgaagtccgacggcttcgccaacagaaacttcatgcagctgatccacgacgacagcctgac
ctttaaagaggacatccagaaagcccaggtgtccggccagggcgatagcctgcacgagcacattgccaatctggccggca
gccccgccattaagaagggcatcctgcagacagtgaaggtggtggacgagctcgtgaaagtgatgggccggcacaagccc
gagaacatcgtgatcgaaatggccagagagaaccagaccacccagaagggacagaagaacagccgcgagagaatgaagcg
gatcgaagagggcatcaaagagctgggcagccagatcctgaaagaacaccccgtggaaaacacccagctgcagaacgaga
agctgtacctgtactacctgcagaatgggcgggatatgtacgtggaccaggaactggacatcaaccggctgtccgactac
gatgtggaccatatcgtgcctcagagctttctgaaggacgactccatcgacaacaaggtgctgaccagaagcgacaagaa
ccggggcaagagcgacaacgtgccctccgaagaggtcgtgaagaagatgaagaactactggcggcagctgctgaacgcca
agctgattacccagagaaagttcgacaatctgaccaaggccgagagaggcggcctgagcgaactggataaggccggcttc
atcaagagacagctggtggaaacccggcagatcacaaagcacgtggcacagatcctggactcccggatgaacactaagta
cgacgagaatgacaagctgatccgggaagtgaaagtgatcaccctgaagtccaagctggtgtccgatttccggaaggatt
tccagttttacaaagtgcgcgagatcaacaactaccaccacgcccacgacgcctacctgaacgccgtcgtgggaaccgcc
ctgatcaaaaagtaccctaagctggaaagcgagttcgtgtacggcgactacaaggtgtacgacgtgcggaagatgatcgc
caagagcgagcaggaaatcggcaaggctaccgccaagtacttcttctacagcaacatcatgaactttttcaagaccgaga
ttaccctggccaacggcgagatccggaagcggcctctgatcgagacaaacggcgaaaccggggagatcgtgtgggataag
ggccgggattttgccaccgtgcggaaagtgctgagcatgccccaagtgaatatcgtgaaaaagaccgaggtgcagacagg
cggcttcagcaaagagtctatcctgcccaagaggaacagcgataagctgatcgccagaaagaaggactgggaccctaaga
agtacggcggcttcgacagccccaccgtggcctattctgtgctggtggtggccaaagtggaaaagggcaagtccaagaaa
ctgaagagtgtgaaagagctgctggggatcaccatcatggaaagaagcagcttcgagaagaatcccatcgactttctgga
agccaagggctacaaagaagtgaaaaaggacctgatcatcaagctgcctaagtactccctgttcgagctggaaaacggcc
ggaagagaatgctggcctctgccggcgaactgcagaagggaaacgaactggccctgccctccaaatatgtgaacttcctg
tacctggccagccactatgagaagctgaagggctcccccgaggataatgagcagaaacagctgtttgtggaacagcacaa
gcactacctggacgagatcatcgagcagatcagcgagttctccaagagagtgatcctggccgacgctaatctggacaaag
tgctgtccgcctacaacaagcaccgggataagcccatcagagagcaggccgagaatatcatccacctgtttaccctgacc
aatctgggagcccctgccgccttcaagtactttgacaccaccatcgaccggaagaggtacaccagcaccaaagaggtgct
ggacgccaccctgatccaccagagcatcaccggcctgtacgagacacggatcgacctgtctcagctgggaggcgacaaaa
ggccggcggccacgaaaaaggccggccaggcaaaaaagaaaaaggaattcggcagtggagagggcagaggaagtctgcta

acatgcggtgacgtcgaggagaatcctggcccaatggtgagcaagggcgaggaggataacatggccatcatcaaggagtt

catgcgcttcaaggtgcacatggagggctccgtgaacggccacgagttcgagatcgagggcgagggcgagggccgcccct

acgagggcacccagaccgccaagctgaaggtgaccaagggtggcccccctgcccttcgcctgggacatcctgtcccctcag

ttcatgtacggctccaaggcctacgtgaagcaccccgccgacatccccgactacttgaagctgtccttccccgagggctt

caagtgggagcgcgtgatgaacttcgaggacggcggcgtggtgaccgtgacccaggactcctccctgcaggacggcgagt

tcatctacaaggtgaagctgcgcggcaccaacttcccctccgacggccccgtaatgcagaagaagaccatgggctgggag

gcctcctccgagcggatgtaccccgaggacggcgccctgaagggcgagatcaagcagaggctgaagctgaaggacggcgg

ccactacgacgctgaggtcaagaccacctacaaggccaagaagcccgtgcagctgcccggcgcctacaacgtcaacatca

agttggacatcacctcccacaacgaggactacaccatcgtggaacagtacgaacgcgccgagggccgccactccaccggc

ggcatggacgagctgtacaagtaggaattctaactagagctcgctgatcagcctcgactgtgccttctagttgccagcca

tctgttgtttgcccctcccccgtgccttccttgaccctggaaggtgccactcccactgtcctttcctaataaaatgagga

aattgcatcgcattgtctgagtaggtgtcattctattctggggggtggggtggggcaggacagcaagggggaggattggg

aagagaatagcaggcatgctggggagcggccgcaggaacccctagtgatggagttggccactccctctctgcgcgctcgc

tcgctcactgaggccgggcgaccaaaggtcgcccgacgcccgggctttgcccgggcggcctcagtgagcgagcgagcgcg

cagctgcctgcaggggcgcctgatgcggtattttctccttacgcatctgtgcggtatttcacaccgcatacgtcaaagca

accatagt

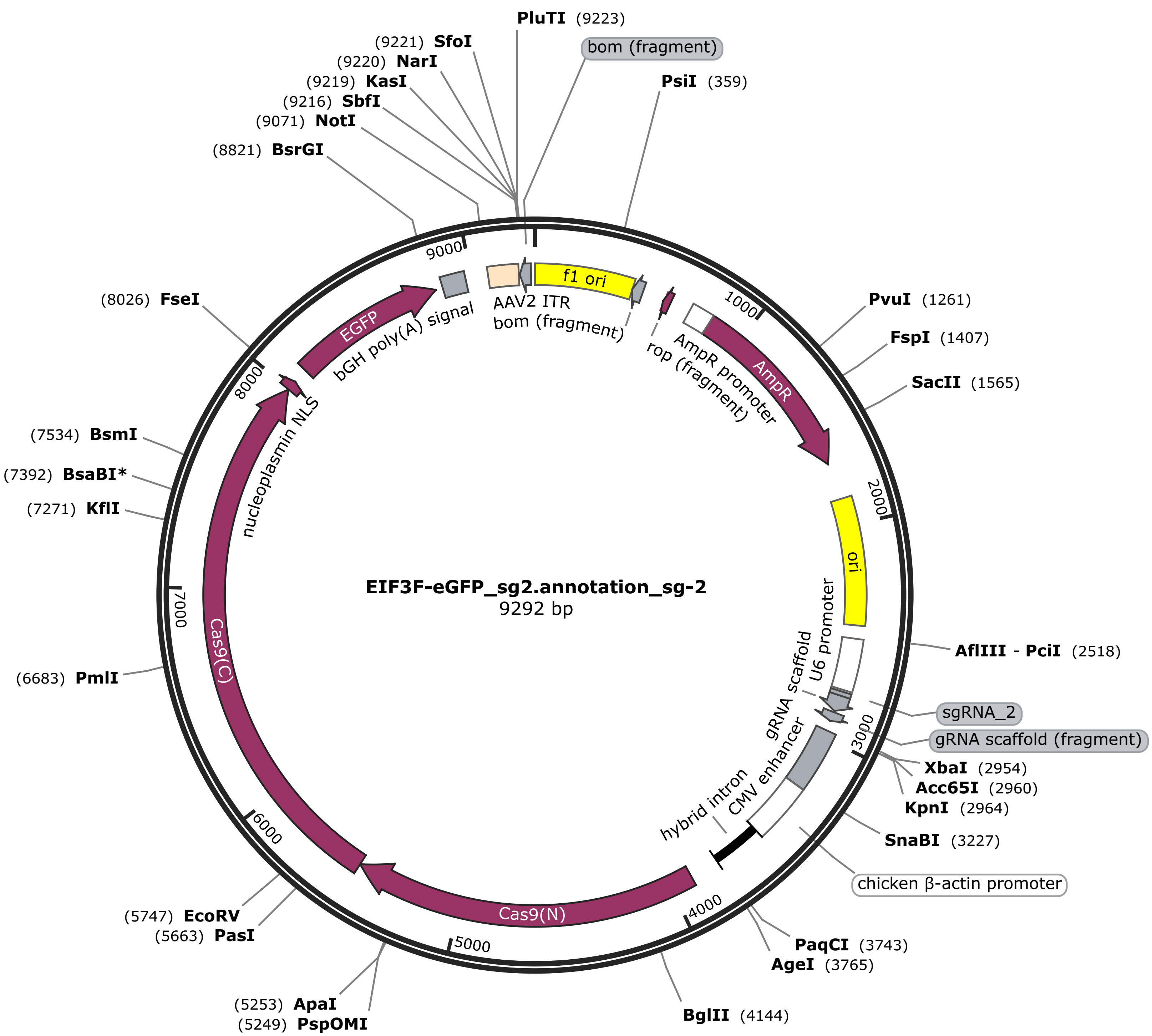
Created by SnapGene
EIF3F-eGFP_sg2.annotation_sg-2
9292 bp
PluTI (9223)
(9221) SfoI
(9220) NarI
(9219) KasI
(9216) SbfI
(9071) NotI
(8821) BsrGI
bom (fragment)
PsiI (359)
PvuI (1261)
FspI (1407)
SacII (1565)
(8026) FseI
(7534) BsmI
(7392) BsaBI*
(7271) KflI
(6683) PmlI
AflIII - PciI (2518)
sgRNA_2
gRNA scaffold (fragment)
XbaI (2954)
Acc65I (2960)
KpnI (2964)
SnaBI (3227)
chicken β-actin promoter
PaqCI (3743)
AgeI (3765)
BglII (4144)
(5747) EcoRV
(5663) PasI
(5253) ApaI
(5249) PspOMI
f1 ori
AAV2 ITR
bom (fragment)
rop (fragment)
AmpR promoter
AmpR
ori
U6 promoter
gRNA scaffold
CMV enhancer
hybrid intron
Cas9(N)
Cas9(C)
nucleoplasmin NLS
EGFP
bGH poly(A) signal
1000
2000
3000
4000
5000
6000
7000
8000
9000

EIF3F-eGFP_sg2.annotation_sg-2.dna

acgcgccctgtagcggcgcattaagcgcggcgggtgtggtggttacgcgcagcgtgaccgctacacttgccagcgccctta

gcgcccgctcctttcgctttcttcccttcctttctcgccacgttcgccggctttccccgtcaagctctaaatcgggggct

ccctttagggttccgatttagtgctttacggcacctcgaccccaaaaaacttgatttgggtgatggttcacgtagtgggc

catcgccctgatagacggtttttcgccctttgacgttggagtccacgttctttaatagtggactcttgttccaaactgga

acaacactcaactctatctcgggctattcttttgatttataagggattttgccgatttcggtctattggttaaaaaatga

gctgatttaacaaaaatttaacgcgaattttaacaaaatattaacgtttacaattttatggtgcactctcagtacaatct

gctctgatgccgcatagttaagccagccccgacacccgccaacacccgctgacgcgccctgacgggcttgtctgctcccg

gcatccgcttacagacaagctgtgaccgtctccgggagctgcatgtgtcagaggttttcaccgtcatcaccgaaacgcgc

gagacgaaagggcctcgtgatacgcctatttttataggttaatgtcatgataataatggtttcttagacgtcaggtggca

cttttcggggaaatgtgcgcggaacccctatttgtttatttttctaaatacattcaaatatgtatccgctcatgagacaa

taaccctgataaatgcttcaataatattgaaaaaggaagagtatgagtattcaacatttccgtgtcgcccttattccctt

ttttgcggcattttgccttcctgtttttgctcacccagaaacgctggtgaaagtaaaagatgctgaagatcagttgggtg

cacgagtgggttacatcgaactggatctcaacagcggtaagatccttgagagttttcgccccgaagaacgttttccaatg

atgagcacttttaaagttctgctatgtggcgcggtattatcccgtattgacgccgggcaagagcaactcggtcgccgcat

acactattctcagaatgacttggttgagtactcaccagtcacagaaaagcatcttacggatggcatgacagtaagagaat

tatgcagtgctgccataaccatgagtgataacactgcggccaacttacttctgacaacgatcggaggaccgaaggagcta

accgcttttttgcacaacatgggggatcatgtaactcgccttgatcgttgggaaccggagctgaatgaagccataccaaa

cgacgagcgtgacaccacgatgcctgtagcaatggcaacaacgttgcgcaaactattaactggcgaactacttactctag

cttcccggcaacaattaatagactggatggaggcggataaagttgcaggaccacttctgcgctcggcccttccggctggc

tggtttattgctgataaatctggagccggtgagcgtggaagccgcggtatcattgcagcactggggccagatggtaagcc

ctcccgtatcgtagttatctacacgacggggagtcaggcaactatggatgaacgaaatagacagatcgctgagataggtg

cctcactgattaagcattggtaactgtcagaccaagtttactcatatatactttagattgatttaaaacttcatttttaa

tttaaaaggatctaggtgaagatcctttttgataatctcatgaccaaaatcccttaacgtgagttttcgttccactgagc

gtcagaccccgtagaaaagatcaaaggatcttcttgagatcctttttttctgcgcgtaatctgctgcttgcaaacaaaaa

aaccaccgctaccagcggtggtttgtttgccggatcaagagctaccaactctttttccgaaggtaactggcttcagcaga

gcgcagataccaaatactgttcttctagtgtagccgtagttaggccaccacttcaagaactctgtagcaccgcctacata

cctcgctctgctaatcctgttaccagtggctgctgccagtggcgataagtcgtgtcttaccgggttggactcaagacgat

agttaccggataaggcgcagcggtcgggctgaacggggggttcgtgcacacagcccagcttggagcgaacgacctacacc

gaactgagatacctacagcgtgagctatgagaaagcgccacgcttcccgaagggagaaaggcggacaggtatccggtaag

cggcagggtcggaacaggagagcgcacgagggagcttccagggggaaacgcctggtatctttatagtcctgtcgggtttc

gccacctctgacttgagcgtcgatttttgtgatgctcgtcaggggggcggagcctatggaaaaacgccagcaacgcggcc

tttttacggttcctggccttttgctggccttttgctcacatgtgagggcctatttcccatgattccttcatatttgcata

tacgatacaaggctgttagagagataattggaattaatttgactgtaaacacaaagatattagtacaaaatacgtgacgt

agaaagtaataatttcttgggtagtttgcagttttaaaattatgttttaaaatggactatcatatgcttaccgtaacttg
aaagtatttcgatttcttggctttatatatcttgtggaaaggacgaaacaccgctgatacacttatagactaggttttag
agctagaaatagcaagttaaaataaggctagtccgttatcaacttgaaaaagtggcaccgagtcggtgctttttttgtttt
agagctagaaatagcaagttaaaataaggctagtccgtttttagcgcgtgcgccaattctgcagacaaatggctctagag
gtacccgttacataacttacggtaaatggcccgcctggctgaccgcccaacgacccccgcccattgacgtcaatagtaac
gccaatagggactttccattgacgtcaatgggtggagtatttacggtaaactgcccacttggcagtacatcaagtgtatc
atatgccaagtacgccccctattgacgtcaatgacggtaaatggcccgcctggcattgtgcccagtacatgaccttatgg
gactttcctacttggcagtacatctacgtattagtcatcgctattaccatggtcgaggtgagccccacgttctgcttcac
tctccccatctcccccccctccccacccccaattttgtatttatttattttttaattattttgtgcagcgatgggggcg
ggggggggggggggggcgcgcgccaggcggggcggggcggggcgaggggcggggcggggcgaggcggagaggtgcggcggc
agccaatcagagcggcgcgctccgaaagtttccttttatggcgaggcggcggcggcggcggccctataaaaagcgaagcg
cgcggcgggcgggagtcgctgcgcgctgccttcgccccgtgccccgctccgccgccgcctcgcgccgcccgccccggctc
tgactgaccgcgttactcccacaggtgagcgggcgggacggcccttctcctccgggctgtaattagctgagcaagaggta
agggtttaagggatggttggttggtggggtattaatgtttaattacctggagcacctgcctgaaatcactttttttcagg
ttggaccggtgccaccatggactataaggaccacgacggagactacaaggatcatgatattgattacaaagacgatgacg
ataagatggccccaaagaagaagcggaaggtcggtatccacggagtcccagcagccgacaagaagtacagcatcggcctg
gacatcggcaccaactctgtgggctgggccgtgatcaccgacgagtacaaggtgcccagcaagaaattcaaggtgctggg
caacaccgaccggcacagcatcaagaagaacctgatcggagccctgctgttcgacagcggcgaaacagccgaggccaccc
ggctgaagagaaccgccagaagaagatacaccagacggaagaaccggatctgctatctgcaagagatcttcagcaacgag
atggccaaggtggacgacagcttcttccacagactggaagagtccttcctggtggaagaggataagaagcacgagcggca
ccccatcttcggcaacatcgtggacgaggtggcctaccacgagaagtaccccaccatctaccacctgagaaagaaactgg
tggacagcaccgacaaggccgacctgcggctgatctatctggccctggcccacatgatcaagttccggggccacttcctg
atcgagggcgacctgaaccccgacaacagcgacgtggacaagctgttcatccagctggtgcagacctacaaccagctgtt
cgaggaaaaccccatcaacgccagcggcgtggacgccaaggccatcctgtctgccagactgagcaagagcagacggctgg
aaaatctgatcgcccagctgcccggcgagaagaagaatggcctgttcggaaacctgattgccctgagcctgggcctgacc
cccaacttcaagagcaacttcgacctggccgaggatgccaaactgcagctgagcaaggacacctacgacgacgacctgga
caacctgctggcccagatcggcgaccagtacgccgacctgtttctggccgccaagaacctgtccgacgccatcctgctga
gcgacatcctgagagtgaacaccgagatcaccaaggcccccctgagcgcctctatgatcaagagatacgacgagcaccac
caggacctgaccctgctgaaagctctcgtgcggcagcagctgcctgagaagtacaaagagattttcttcgaccagagcaa
gaacggctacgccggctacattgacggcggagccagccaggaagagttctacaagttcatcaagcccatcctggaaaaga
tggacggcaccgaggaactgctcgtgaagctgaacagagaggacctgctgcggaagcagcggaccttcgacaacggcagc
atccccaccagatccacctgggagagctgcacgccattctgcggcggcaggaagatttttacccattcctgaaggacaa
ccgggaaaagatcgagaagatcctgaccttccgcatcccctactacgtgggccctctggccaggggaaacagcagattcg
cctggatgaccagaaagagcgaggaaaccatcacccccctggaacttcgaggaagtggtggacaagggcgcttccgcccag

agcttcatcgagcggatgaccaacttcgataagaacctgcccaacgagaaggtgctgcccaagcacagcctgctgtacga
gtacttcaccgtgtataacgagctgaccaaagtgaaatacgtgaccgagggaatgagaaagcccgccttcctgagcggcg
agcagaaaaaggccatcgtggacctgctgttcaagaccaaccggaaagtgaccgtgaagcagctgaaagaggactacttc
aagaaaatcgagtgcttcgactccgtggaaatctccggcgtggaagatcggttcaacgcctccctgggcacataccacga
tctgctgaaaattatcaaggacaaggacttcctggacaatgaggaaaacgaggacattctggaagatatcgtgctgaccc
tgacactgtttgaggacagagagatgatcgaggaacggctgaaaacctatgcccacctgttcgacgacaaagtgatgaag
cagctgaagcggcggagatacaccggctggggcaggctgagccggaagctgatcaacggcatccgggacaagcagtccgg
caagacaatcctggatttcctgaagtccgacggcttcgccaacagaaacttcatgcagctgatccacgacgacagcctga
cctttaaagaggacatccagaaagcccaggtgtccggccagggcgatagcctgcacgagcacattgccaatctggccggc
agccccgccattaagaagggcatcctgcagacagtgaaggtggtggacgagctcgtgaaagtgatgggccggcacaagcc
cgagaacatcgtgatcgaaatggccagagagaaccagaccacccagaagggacagaagaacagccgcgagagaatgaagc
ggatcgaagagggcatcaaagagctgggcagccagatcctgaaagaacaccccgtggaaaacacccagctgcagaacgag
aagctgtacctgtactacctgcagaatgggcgggatatgtacgtggaccaggaactggacatcaaccggctgtccgacta
cgatgtggaccatatcgtgcctcagagctttctgaaggacgactccatcgacaacaaggtgctgaccagaagcgacaaga
accggggcaagagcgacaacgtgccctccgaagaggtcgtgaagaagatgaagaactactggcggcagctgctgaacgcc
aagctgattacccagagaaagttcgacaatctgaccaaggccgagagaggcggcctgagcgaactggataaggccggctt
catcaagagacagctggtggaaacccggcagatcacaaagcacgtggcacagatcctggactcccggatgaacactaagt
acgacgagaatgacaagctgatccgggaagtgaaagtgatcaccctgaagtccaagctggtgtccgatttccggaaggat
ttccagttttacaaagtgcgcgagatcaacaactaccaccacgcccacgacgcctacctgaacgccgtcgtgggaaccgc
cctgatcaaaaagtaccctaagctggaaagcgagttcgtgtacggcgactacaaggtgtacgacgtgcggaagatgatcg
ccaagagcgagcaggaaatcggcaaggctaccgccaagtacttcttctacagcaacatcatgaactttttcaagaccgag
attaccctggccaacggcgagatccggaagcggcctctgatcgagacaaacggcgaaaccggggagatcgtgtgggataa
gggccgggattttgccaccgtgcggaaagtgctgagcatgccccaagtgaatatcgtgaaaaagaccgaggtgcagacag
gcggcttcagcaaagagtctatcctgcccaagaggaacagcgataagctgatcgccagaaagaaggactgggaccctaag
aagtacggcggcttcgacagccccaccgtggcctattctgtgctggtggtggccaaagtggaaaagggcaagtccaagaa
actgaagagtgtgaaagagctgctggggatcaccatcatggaaagaagcagcttcgagaagaatcccatcgactttctgg
aagccaagggctacaaagaagtgaaaaaggacctgatcatcaagctgcctaagtactccctgttcgagctggaaaacggc
cggaagagaatgctggcctctgccggcgaactgcagaagggaaacgaactggccctgccctccaaatatgtgaacttcct
gtacctggccagccactatgagaagctgaagggctcccccgaggataatgagcagaaacagctgtttgtggaacagcaca
agcactacctggacgagatcatcgagcagatcagcgagttctccaagagagtgatcctggccgacgctaatctggacaaa
gtgctgtccgcctacaacaagcaccgggataagcccatcagagagcaggccgagaatatcatccacctgtttaccctgac
caatctgggagcccctgccgccttcaagtactttgacaccaccatcgaccggaagaggtacaccagcaccaaagaggtgc
tggacgccaccctgatccaccagagcatcaccggcctgtacgagacacggatcgacctgtctcagctgggaggcgacaaa
aggccggcggccacgaaaaaggccggccaggcaaaaaagaaaaaggaattcggcagtggagagggcagaggaagtctgct

aacatgcggtgacgtcgaggagaatcctggcccagtgagcaagggcgaggagctgttcaccggggtggtgcccatcctgg
tcgagctggacggcgacgtaaacggccacaagttcagcgtgtccggcgagggcgagggcgatgccacctacggcaagctg
accctgaagttcatctgcaccaccggcaagctgcccgtgccctggcccaccctcgtgaccaccctgacctacggcgtgca
gtgcttcagccgctaccccgaccacatgaagcagcacgacttcttcaagtccgccatgcccgaaggctacgtccaggagc
gcaccatcttcttcaaggacgacggcaactacaagacccgcgccgaggtgaagttcgagggcgacaccctggtgaaccgc
atcgagctgaagggcatcgacttcaaggaggacggcaacatcctggggcacaagctggagtacaactacaacagccacaa
cgtctatatcatggccgacaagcagaagaacggcatcaaggtgaacttcaagatccgccacaacatcgaggacggcagcg
tgcagctcgccgaccactaccagcagaacacccccatcggcgacggccccgtgctgctgcccgacaaccactacctgagc
acccagtccgccctgagcaaagaccccaacgagaagcgcgatcacatggtcctgctggagttcgtgaccgccgccgggat
cactctcggcatggacgagctgtacaaggaattctaactagagctcgctgatcagcctcgactgtgccttctagttgcca
gccatctgttgtttgcccctcccccgtgccttccttgaccctggaaggtgccactcccactgtcctttcctaataaaatg
aggaaattgcatcgcattgtctgagtaggtgtcattctattctggggggtggggtggggcaggacagcaagggggaggat
tgggaagagaatagcaggcatgctggggagcggccgcaggaacccctagtgatggagttggccactccctctctgcgcgc
tcgctcgctcactgaggccgggcgaccaaaggtcgcccgacgcccgggctttgcccgggcggcctcagtgagcgagcgag
cgcgcagctgcctgcaggggcgcctgatgcggtattttctccttacgcatctgtgcggtatttcacaccgcatacgtcaa
agcaaccatagt
